# Changes in the concentrations and transcripts for gibberellins and other hormones in a growing leaf and roots of wheat seedlings in response to water restriction

**DOI:** 10.1186/s12870-022-03667-w

**Published:** 2022-06-09

**Authors:** Klára Ptošková, Marek Szecówka, Pavel Jaworek, Danuše Tarkowská, Ivan Petřík, Iva Pavlović, Ondřej Novák, Stephen G. Thomas, Andrew L. Phillips, Peter Hedden

**Affiliations:** 1 Laboratory of Growth Regulators, Institute of Experimental Botany, Czech Academy of Sciences and Palacky University, Šlechtitelů 27, CZ-78371 Olomouc, Czech Republic; 2 Department of Plant Science, Rothamsted Research, Harpenden, AL5 2JQ UK

**Keywords:** Drought, gene expression, gibberellins, plant hormones, wheat

## Abstract

**Background:**

Bread wheat (*Triticum aestivum*) is a major source of nutrition globally, but yields can be seriously compromised by water limitation. Redistribution of growth between shoots and roots is a common response to drought, promoting plant survival, but reducing yield. Gibberellins (GAs) are necessary for shoot and root elongation, but roots maintain growth at lower GA concentrations compared with shoots, making GA a suitable hormone for mediating this growth redistribution. In this study, the effect of progressive drought on GA content was determined in the base of the 4th leaf and root tips of wheat seedlings, containing the growing regions, as well as in the remaining leaf and root tissues. In addition, the contents of other selected hormones known to be involved in stress responses were determined. Transcriptome analysis was performed on equivalent tissues and drought-associated differential expression was determined for hormone-related genes.

**Results:**

After 5 days of applying progressive drought to 10-day old seedlings, the length of leaf 4 was reduced by 31% compared with watered seedlings and this was associated with significant decreases in the concentrations of bioactive GA_1_ and GA_4_ in the leaf base, as well as of their catabolites and precursors. Root length was unaffected by drought, while GA concentrations were slightly, but significantly higher in the tips of droughted roots compared with watered plants. Transcripts for the GA-inactivating gene *TaGA2ox4* were elevated in the droughted leaf, while those for several GA-biosynthesis genes were reduced by drought, but mainly in the non-growing region. In response to drought the concentrations of abscisic acid, *cis-*zeatin and its riboside increased in all tissues, indole-acetic acid was unchanged, while *trans*-zeatin and riboside, jasmonate and salicylic acid concentrations were reduced.

**Conclusions:**

Reduced leaf elongation and maintained root growth in wheat seedlings subjected to progressive drought were associated with attenuated and increased GA content, respectively, in the growing regions. Despite increased *TaGA2ox4* expression, lower GA levels in the leaf base of droughted plants were due to reduced biosynthesis rather than increased catabolism. In contrast to GA, the other hormones analysed responded to drought similarly in the leaf and roots, indicating organ-specific differential regulation of GA metabolism in response to drought.

**Supplementary Information:**

The online version contains supplementary material available at 10.1186/s12870-022-03667-w.

## Background

Wheat (*Triticum aestivum* L.) is one of the most important staple crops globally feeding a major part of the human population. The worldwide estimated production of wheat in 2019 was 766 million tonnes making it the second most important grain after maize [1]. However, drought is a major constraint to the productivity of wheat and other cereals [2–4] and is anticipated to become an even more serious problem for farmers worldwide as a result of the changing climate [5, 6].

Plants under drought stress adapt their morphology, physiology and biochemistry in an attempt to cope with the water limitation [7–9]. Plant hormones are important components of this stress response, mediating mechanisms to reduce water usage, including restriction of shoot growth, while roots, which first perceive the lack of water, continue to extend [10–12]. Gibberellins (GAs) as growth regulators are prime candidates for involvement in this growth redistribution. Furthermore, it has been shown that plants with reduced GA content, through the application of growth retardants or mutation, are more resistant to abiotic stress, including drought, cold and salt stress [13]. The mechanism of this phenomenon is still not fully understood.

Gibberellins signal through the GID1 receptor, which, in the presence of GAs, interacts with and destabilises DELLA proteins, a family of transcriptional regulators, first identified as repressors of growth [14]. DELLA proteins act in association with transcription factors to modify gene expression, for example by sequestration of the transcription factors or by functioning as a co-activator [15–17]. After GA-dependant binding to GID1, DELLA proteins are polyubiquitinated by an E3 ubiquitin ligase and targeted for degradation by the 26S proteasome [18, 19]. In rice, barley and Arabidopsis DELLA protein loss-of-function mutants have been shown to exhibit a constitutive GA response phenotype [20, 21]. These mutants do not respond to exogenous GA, are taller than the wild-type, flower early and are often sterile. On the other hand, specific mutations in the N-terminal region of DELLA proteins cause their accumulation by preventing their association with the GA-GID1 complex [20–24]. Such gain-of-function mutants are dwarfed; prime examples of which are the semi-dwarfing alleles of the wheat *RHT-1* gene, which were introduced in the Green Revolution and are present in most commercial cultivars [25]. Wheat cultivars containing *RHT-1* dwarfing alleles were found to perform better under water deprivation than those carrying the non-mutant tall allele [26].

During abiotic stress, DELLA proteins accumulate in association with a reduction in GA content [27, 28]. As well as decreasing water requirement through suppressing shoot growth, the accumulation of DELLA was shown to reduce transpiration in tomato by closing stomata [29]. The mechanism involves increased ABA concentration through enhanced expression of an ABA transporter [30], highlighting the action of DELLA as a hub in the cross-talk between the GA signalling pathway and those of other plant hormones [31]. In addition, DELLA was observed to reduce the level of reactive oxygen species [27], which accumulate during abiotic stress and can lead to oxidative damage and cell death when present at high concentrations [32].

Stress-induced DELLA accumulation is enabled primarily by a reduction in GA concentration, although expression of some DELLA paralogues, such as *RGL3* in Arabidopsis, is up-regulated by stress [33, 34]. Biosynthesis of GAs proceeds through the action of plasmid-localised terpene cyclases, *ent*-copalyl diphosphate synthase (CPS) and *ent*-kaurene synthase (KS), and the membrane-associated cytochrome P450 monooxygenases, *ent*-kaurene oxidase (KO) and *ent*-kaurenoic acid oxidase (KAO) to produce the C_20_-GA GA_12_, which is 13-hydroxylated by cytochrome P450 monooxygenases (GA13ox) to GA_53_ [35]. GA_12_ and GA_53_ are converted by the soluble 2-oxoglutarate-dependent dioxygenases GA 20-oxidase (GA20ox) and GA 3-oxidase (GA3ox) to the biologically active C_19_-GA end-products GA_4_ and GA_1_, respectively. GA turnover occurs primarily through the action of the inactivating GA 2-oxidases (GA2ox), of which there are two families, acting on C_19_-GAs or C_20_-GAs, respectively [36–39]. Up-regulation of *GA2ox* genes to reduce GA content and promote DELLA accumulation has been shown to occur in response to abiotic stress, with examples from Arabidopsis for up-regulation of the *AtGA2ox7* paralogue in response to salt [40] and mechanical stimulus [41], and of several *GA2ox* genes in response to cold [33, 36]. Pearce et al. [42] identified 7 *C*_*19*_*-GA2ox* paralogues, excluding homoeologues, and 5 *C*_*20*_*-GA2ox* paralogues in the wheat genome but there is limited information on their involvement in stress responses for this species.

While the growth of shoots and roots is dependent on GA signalling, root growth requires lower concentrations of GAs than does that of shoots [43] and indeed root growth may be inhibited by supraoptimum GA concentrations [44, 45]. Thus, a reduction in GA content in response to drought provides a mechanism to redistribute growth between shoots and roots, but the role of GA signalling in this response has been little studied. In this report, we describe the effect of water limitation on the growth, transcriptome and hormone content of shoots and roots of wheat seedlings with a focus on GA metabolism and signalling.

## Results

### The effect of water restriction on growth and physiology

**Fig. 1:**
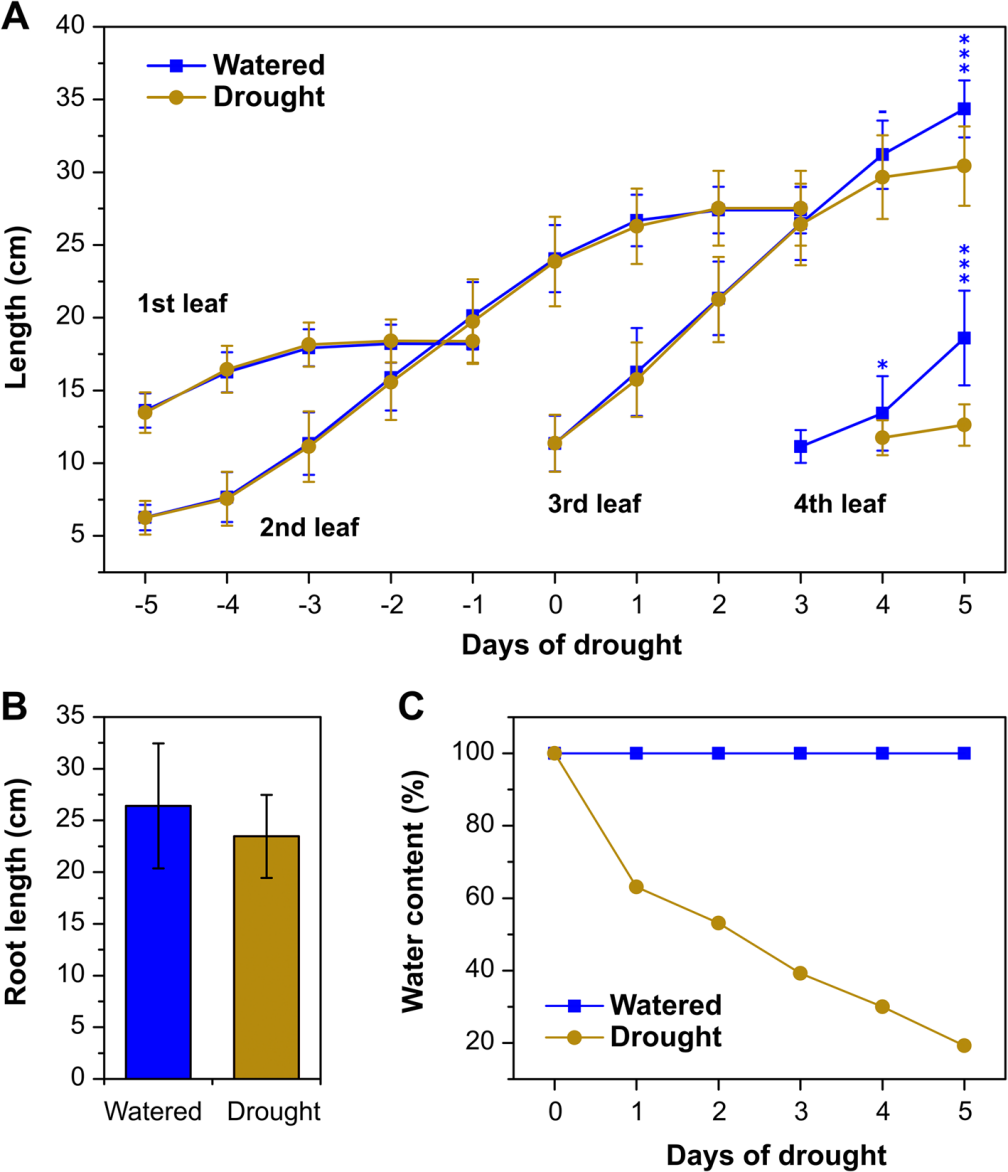
Growth response of wheat seedlings to prolonged drought. **A** Mean lengths +/− SD of leaves 1-4 measured from 5 days before and after the cessation of watering. Statistically significant differences between watered and droughted plants are indicated by * (*p* < 0.05) and *** (*p* < 0.001), *n* = 20. **B** Mean length of the longest root +/− SD at 5 days after water cessation, n = 20. **C** Soil water content (% full soil water capacity)

Wheat seedlings were grown in pots under a controlled environment in a field soil with a high sand content until the third leaf was visible. Watering was then withheld from half the plants, while the remainder were continued to be watered to 100% soil capacity. Leaf lengths were monitored throughout the experiment (Fig. 1A). After 5 days, the length of leaf 4 in the droughted plants was reduced by 31% compared with the watered plants, while the lengths of the longest root were not significantly different between the two treatments (Fig. 1B). At this stage, the water content of the droughted soil was 19% full capacity (Fig. 1C), and the relative water content of the 3rd leaf was reduced from 96% for the well-watered plants to 81.5% under drought (Additional file 1: Table S1). The contents of malondialdehyde (MDA), indicative of lipid oxidation, and proline in leaves of the droughted seedlings increased by 2- and 5-fold, respectively, compared with the well-watered seedlings (Additional file 1: Table S1). There was a small, but non-significant (*p* = 0.2) reduction in photosynthetic rate in the droughted seedlings after 5 days of water restriction compared with the well-watered plants, while by 6 days the difference was significant (*p* = 0.04) (Additional file 1: Table S1). Similarly, there were small, non-significant reductions in stomatal conductance and intercellular CO_2_ concentrations on day 5 for the droughted plants with greater, significant reductions after 6 days (Additional file 1: Table S1). In order to determine changes in hormone content and gene expression in response to water restriction, the 4th leaf was harvested at day 5 and divided into the bottom 3 cm of the sheath (leaf base), which includes the growing region of the leaf sheath, and the rest of the leaf. Roots were divided into the bottom 3 cm of the primary roots (the root tip) and the remaining root material (Additional file 1: Fig. S1 illustrates the tissue sampling). The four samples from well-watered and droughted seedlings with replicates were analysed for the abundance of GAs and other hormones by ultra-high performance liquid chromatography tandem mass spectrometry (UHPLC-MS-MS) and of transcripts by RNA-sequencing.

### Water restriction and gibberellin content

**Fig. 2:**
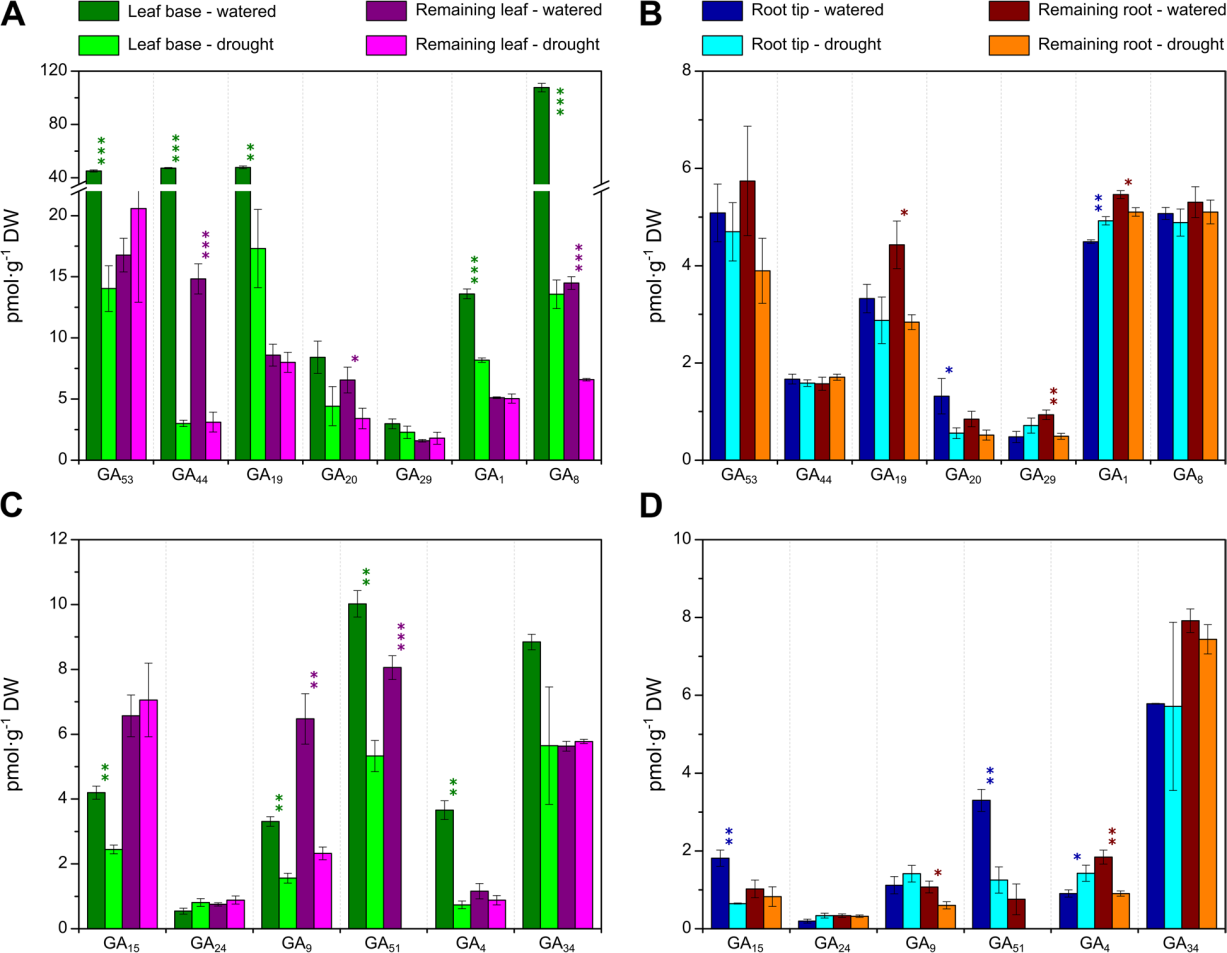
Concentrations of gibberellins (GAs) in the 4th leaf and roots of watered and droughted wheat seedlings after 5 days of water cessation. GAs were analysed in the leaf base and remaining leaf (**A** and **C** for 13-OH- and 13-H-GAs, respectively) and in the root tip and remaining root (**B** and **D**, for 13-OH- and 13-H-GAs, respectively). Values are the means of 3 replicates +/− SD. Statistically significant differences between watered and droughted plants are indicated by * (*p* < 0.05); ** (*p* < 0.01) and *** (*p* < 0.001) determined by the Student t-test

The GAs, including precursors and catabolites, were analysed separately from other hormones due to the requirement for a different protocol in order to achieve adequate sensitivity. Figure 2A and C show the concentrations of 13-hydroxylated (13-OH) and 13-deoxy (13-H) GAs, respectively, in the leaf tissues, while these are presented for the roots in Fig. 2B and D. In the base of the leaf, which contains the extension zone of the sheath [46], the concentrations of most GAs, including the biologically active GA_1_ and GA_4_ were significantly reduced by water restriction. Notably, the concentration of GA_8_, the 2β-hydroxylated catabolite of GA_1_, was also strongly reduced by drought. GA_1_ and GA_4_ contents were unchanged by drought in the remaining leaf, although GA_8_ concentration, but not that of GA_34_ (2β-hydroxyGA_4_), was significantly reduced. The effect of drought on precursor levels in the remaining leaf was variable with only GA_44_ and GA_20_ showing significant decreases. In contrast to the base of the leaf, the root tip showed small, but significant increases in GA_1_ and GA_4_ concentrations under drought, while the levels of GA_15_, GA_20_ and GA_51_ (2β-hydroxyGA_9_) decreased and those of the other GAs remained unchanged. In the remaining root tissue there were small decreases in the levels of several GAs under drought, including GA_1_ and GA_4_ and the precursor GA_19_. Notably, the levels of the major catabolites, GA_8_ and GA_34_, were not changed by drought in either root tissue.

### Water restriction and the concentration of other hormones

**Fig. 3:**
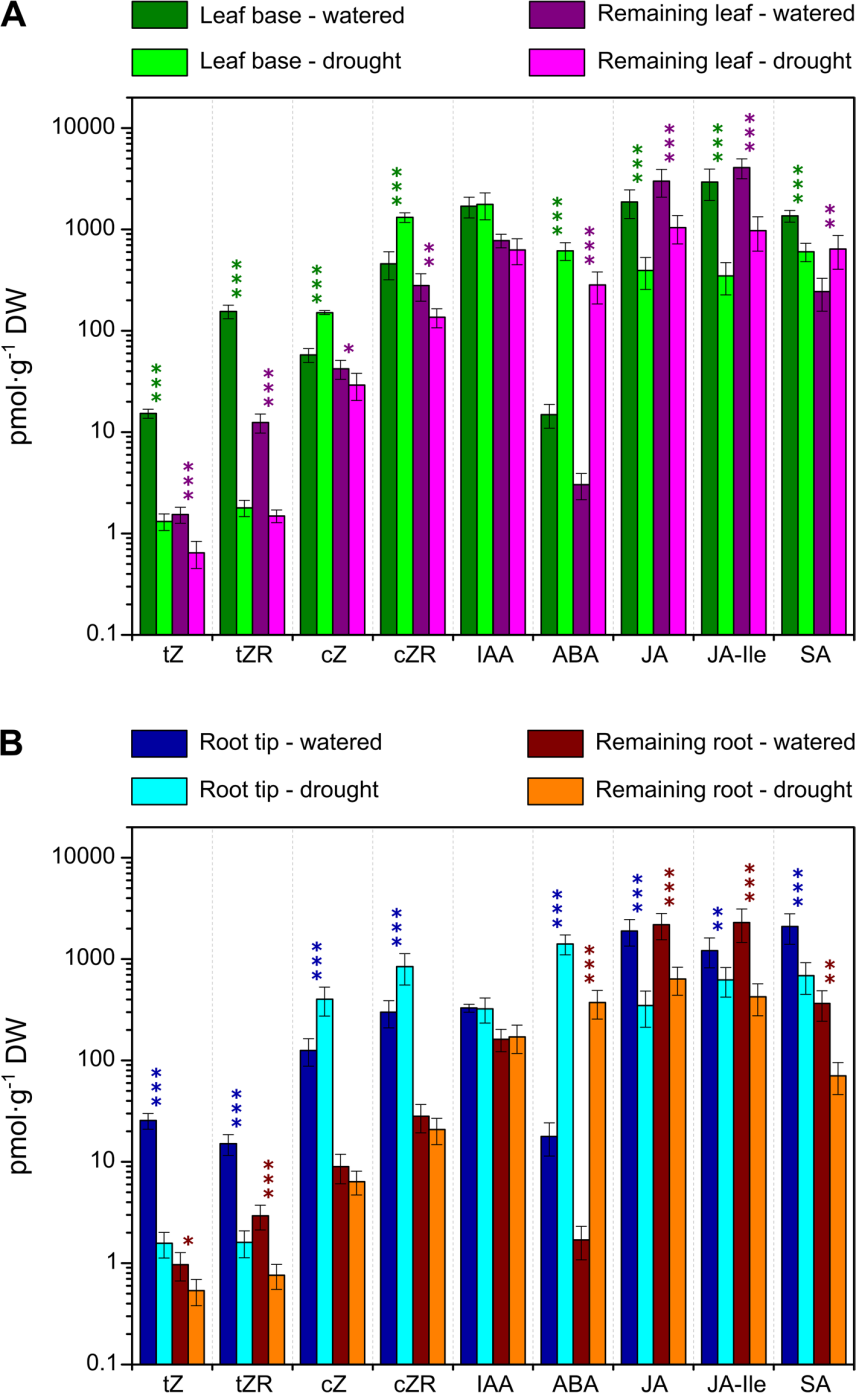
Concentration of selected hormones in the 4th leaf and roots of watered and droughted wheat seedlings after 5 days of water cessation. Concentrations were determined in the leaf base and remaining leaf (**A**) and the root tip and remaining root (**B**). Values are the means of 7 replicates +/− SD. Statistically significant differences between watered and droughted plants are indicated by * (*p* < 0.05); ** (*p* < 0.01) and *** (*p* < 0.001) determined by the Student t-test. *t*Z, *trans*-zeatin; *t*ZR, *trans*-zeatin riboside; *c*Z, *cis*-zeatin; *c*ZR, *cis*-zeatin riboside; IAA, indole-3-acetic acid; ABA, abscisic acid; JA, jasmonic acid; JA-Ile, jasmonic acid isoleucine conjugate; SA, salicylic acid

Growth and its response to water limitation are regulated by the combined activity of multiple hormones [47, 48], so we took the opportunity to measure the concentrations of abscisic acid (ABA), indole-3-acetic acid (IAA), cytokinins (CKs), jasmonates (JAs), salicylic acid (SA) and associated metabolites in equivalent samples to those analysed for GAs. Their concentrations are given in Additional file 2, and for selected hormones shown in Fig. 3A and B for the leaf and root samples, respectively. As expected, the concentration of ABA was strongly increased by water limitation in all four tissues, with fold increases of 41 and 94 in the leaf base and remaining leaf, respectively, and of 79 and 219 for the root tip and remaining root tissue, respectively. There were smaller increases under drought in the concentration of the ABA-catabolite phaseic acid in the leaf tissue and lower root (Additional file 2), while it increased more substantially in the remaining root. In contrast to that of ABA, the concentration of IAA was not significantly changed by drought in any of the tissues, although there were higher levels of 2-oxindole-3-acetic acid in all tissues and of IAA-glutamate in the leaf base. The concentrations of JA and its isoleucine conjugate decreased in all four water-stressed tissues, as did that of SA in all tissues except the remaining leaf in which it increased, suggesting a redistribution under drought.

The concentrations of *trans*-zeatin (*t*Z) and its riboside (*t*ZR), which were higher in the growing regions of the leaf and root compared with the remaining tissues, were reduced by water limitation in all sampled tissues. The lower *t*Z and *t*ZR concentrations were accompanied by increased levels of their *O*-glucosides in the leaf base and root tip, but not in the remaining tissues. In contrast, the concentrations of *cis*-zeatin (*c*Z) and its riboside *c*ZR, which were generally higher than those of the *trans* isomers, were increased by drought in the leaf base and root tip, whereas it was reduced in the droughted remaining leaf tissue and unchanged in the remaining root tissue, in which concentrations were generally low. The concentrations of the *O*-glucosides of *c*Z and *c*ZR followed the same trend as their aglycones in the leaf base and root, while there were significant decreases in their levels in the remaining root tissues under drought.

### Water restriction and gene expression

Changes in gene expression due to water restriction was determined by RNA-seq from three biological replicates in tissues and treatments equivalent to those used for the hormone determination. The numbers of differentially expressed genes (DEGs) (> 2-fold change) were substantially higher for the leaf tissues (17,298 and 24,343 for the 4th leaf base and remaining leaf, respectively) than for the root (2696 and 8995 for the root tip and remaining root, respectively) (Table 1). The distribution of up- and down-regulated genes is illustrated by the volcano plots in Additional file 1: Fig. S2. As indicated by the dendrogram and principal component analysis plot in Additional file 1: Fig. S3A and Fig. S3B, respectively, there was relatively close sample replication, with the largest discrimination between leaf and roots, followed by leaf tissue types and then by the leaf tissues in response to drought. In contrast to the leaf, there was comparatively little discrimination between the root samples, for tissue type or treatment. The distribution of DEGs between the tissue types is shown in Additional file 1: Fig. S3C. Of the 37,996 DEGs, 89.5% were expressed in the leaf, of which 84.3% (75.5% of the total) were unique to the leaf and 22.4% were expressed in both leaf tissues. The equivalent figures for the root were 9.3% of the total, of which 42.7% (4% of the total) were unique to the roots and 17.1% were expressed in both root tissues. While the number of unique DEGs was 50 and 59% of those expressed in the leaf base and remaining leaf, respectively, it was only 13% for the root tip and 38% for the remaining root. Full lists of genes with mean normalised reads under well-watered and drought conditions and log_2_-fold change (LFC) are presented in Additional files 3, 4, 5 and 6, in which they are ordered by differential expression. A gene ontology analysis of biological function is shown in Additional file 1: Fig. S4 – Fig. S7 for the leaf base, remaining leaf, root tip and remaining root, showing the 30 most significant down- and up-regulated processes, respectively, for each tissue type. Cellular organisation and metabolism are strongly represented in the down-regulated functions, while responses to abiotic stimuli and related metabolism, and to ABA are major up-regulated processes. Annotated genes for hormone metabolism and signalling are listed in Additional file 7, in which their annotation, if not previously published, is based where possible on that of their rice orthologues.

**Table 1:** Numbers of differentially expressed genes (log_2_-fold change between droughted and watered plants > 1 or < 1)

Tissue	Number of differentially expressed genes	
	Up-regulated	Down-regulated
Leaf base	9175	8123
Remaining leaf	9570	14,773
Root tip	1903	793
Remaining root	4103	4892

#### Gibberellin metabolism and signal transduction

**Fig. 4:**
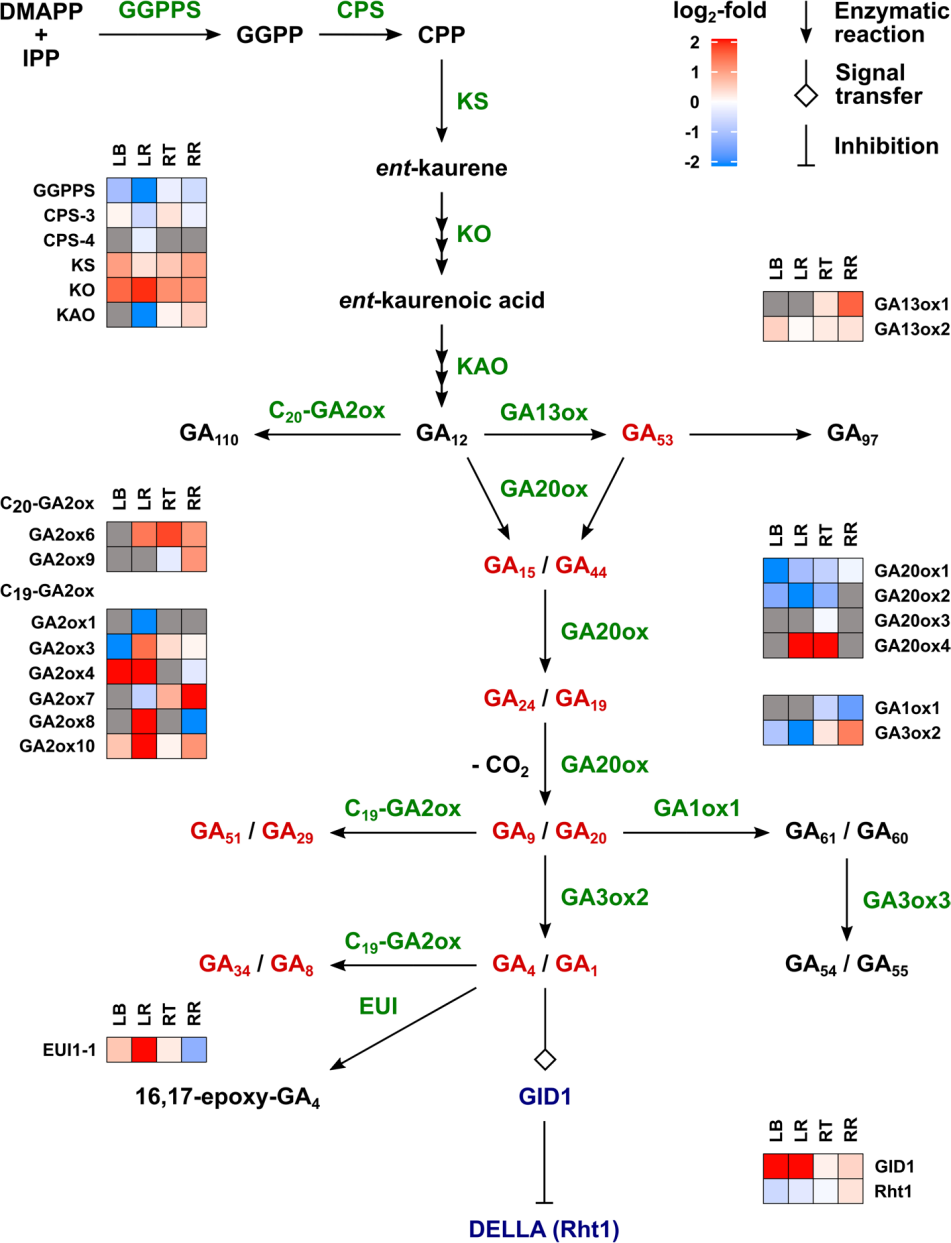
Heat maps for differential expression of genes for GA metabolism and signalling between watered and droughted wheat seedlings in the 4th leaf base and remaining leaf, and root tip and remaining root. For simplicity, the normalized reads for the homoeologues were combined and mean values for three biological replicates were calculated, as described in Methods. The number of normalized reads and LFCs for individual homoeologues together with the gene IDs are provided in Additional file 7. Grey boxes indicate that the number of summed reads are < 10 for the tissue section from both watered and droughted seedlings. Genes for which summed reads are < 10 in each tissue section are omitted. LB, leaf base; LR, remaining leaf; RT, root tip; RR, remaining root

The relative expression in response to drought of genes involved in GA metabolism and signalling is shown for each tissue type as heat maps in Fig. 4. In order to simplify the figure, normalised reads for the three homoeologues are summed, while the normalised reads are provided separately for all homoeologues in Additional file 7. The 2-oxoglutarate-dependent dioxygenase genes of GA biosynthesis and inactivation are considered to be major sites of regulation [35]. Transcripts for *TaGA20ox1* and *2* were more abundant in the remaining leaf tissue than in the leaf base and were reduced by drought in both leaf tissues. There was no significant change due to drought in the root tissues. Notably, there was a relatively high expression of *GA20ox4* in the remaining leaf and, for one homoeologue (*TaGA20ox-A4*) only, expression was increased substantially under drought. Roots contained very few reads for this paralogue, except for the A homoeologue in the droughted root tip. In contrast to the *GA20ox* genes, *GA3ox2* transcripts were evenly distributed between the leaf base and remaining leaf and also in the root tissues. Under drought *GA3ox2* expression was reduced in the leaf, but increased in the root, particularly in the remaining root tissue. Both root tissues contain transcripts for *GA1ox1* that encodes a mildly inactivating enzyme and is highly expressed in developing grain [42]. Its expression was reduced under drought.

For the genes encoding enzymes for the early GA-biosynthetic pathway, *CPS* is present as at least four paralogues in wheat, of which *CPS3* and *CPS4* are reported to be involved in GA biosynthesis [49, 50] and are included in Fig. 4 and Additional file 7. Expression of neither gene is affected by drought in any tissue type. The A homoeologue of *KS* was up-regulated in response to drought in all tissues, while *KO* was up-regulated by drought in the leaf tissues but to a less extent in the roots. *KAO* expression in the leaf was mainly in the remaining leaf tissue, in which it was down-regulated by drought, while it was not differentially regulated by drought in the roots. Transcripts of *trans*-geranylgeranyl diphosphate synthase (*GGPPS*) homoeologues, responsible for diterpene biosynthesis, were reduced by drought in the leaf, particularly in the remaining leaf tissue, but were not affected by drought in the root tissues.

Of the wheat *GA2ox* genes, responsible for GA-turnover by inactivation, the enzymes encoded by *TaGA2ox6* and *TaGA2ox9* act mainly on C_20_-GAs [42], while *TaGA2ox11*, which is orthologous to the rice *GA2ox11*, belongs to the same clade and is assumed also to encode a C_20_-GA2ox [51]. The genes annotated as *TaGA2ox11, 12* and *13* in Pearce et al. [42] are close homologues of *TaGA2ox6* and are renamed as *TaGA2ox6-2, 6-3* and *6-4*, respectively (Additional file 7). The remaining *TaGA2ox* genes encode enzymes that act mainly on C_19_-GAs [42]. No reads were present for *TaGA2ox2*, *TaGA2ox6-4* and *TaGA2ox11* in any of the sampled tissues, while expression of *TaGA2ox1, 6-1, 6-2, 6-3, 8* and *9* was low in all tissues. In both sections of the droughted fourth leaf, there was strong up-regulation of *TaGA2ox4*, with *TaGA2ox3* and *TaGA2ox10* also up-regulated in the remaining leaf, but not in the leaf base. Indeed, *TaGA2ox3* transcripts, which were more abundant in the base of the sheath, were present at lower levels in this tissue in response to drought. In contrast, expression of *TaGA2ox4* under well-watered conditions was higher in the remaining leaf, while water restriction caused strong up-regulation in both tissues, particularly in the leaf base. Expression of *TaGA2ox7* was mainly confined to the remaining leaf, in which two of its homoeologues were down-regulated under drought, whereas *TaGA2ox10* was expressed more highly in the leaf base and was the most highly expressed *GA2ox* gene in this tissue under well-watered conditions. Under drought, expression of *TaGA2ox10* increased in the remaining leaf, but only expression of the D homoeologue increased in the leaf base. In the roots, *TaGA2ox3* was by far the most highly expressed *GA2ox* gene and its expression in both tissue types was not affected by drought.

Genes encoding the GA-receptor GID1 and the DELLA protein RHT-1 were highly expressed in all four tissues. Notably, expression of *GID1* was strongly promoted under drought in the leaf tissues, but not in the roots, while *RHT-1* was not differentially expressed under drought in any tissue. Transcripts for genes encoding the F-box protein GID2, a component of the E3-ligase responsible for RHT-1 degradation [52, 53], are not included in the analysis. *GID2* exists in wheat as two paralogues, of which the closest orthologue to the rice gene (TraesCS3A01G055700 and TraesCS3B01G068100) is present in the intron of another gene and its expression could not be determined accurately. The function of its paralogues (*GID2-like*: TraesCS3A01G056000, TraesCS3A01G511800, TraesCS3B01G068800 and TraesCS3D01G056100) is unclear.

In a separate experiment, differential expression in the four tissue types in response to water restriction was determined by qRT-PCR for three *GA2ox* genes (*GA2ox3*, *GA2ox4* and *GA2ox10*), the biosynthetic gene *GA3ox2* as well as *GID1* and the ABA signalling gene *PP2C1*, which is strongly up-regulated by drought (see below). The results from qRT-PCR, for which primers were designed to amplify all three homoeologues, were generally consistent with the differential expression determined by RNA-seq (Additional file 1: Fig. S8).

#### Metabolism and signalling for other hormones

**Fig. 5:**
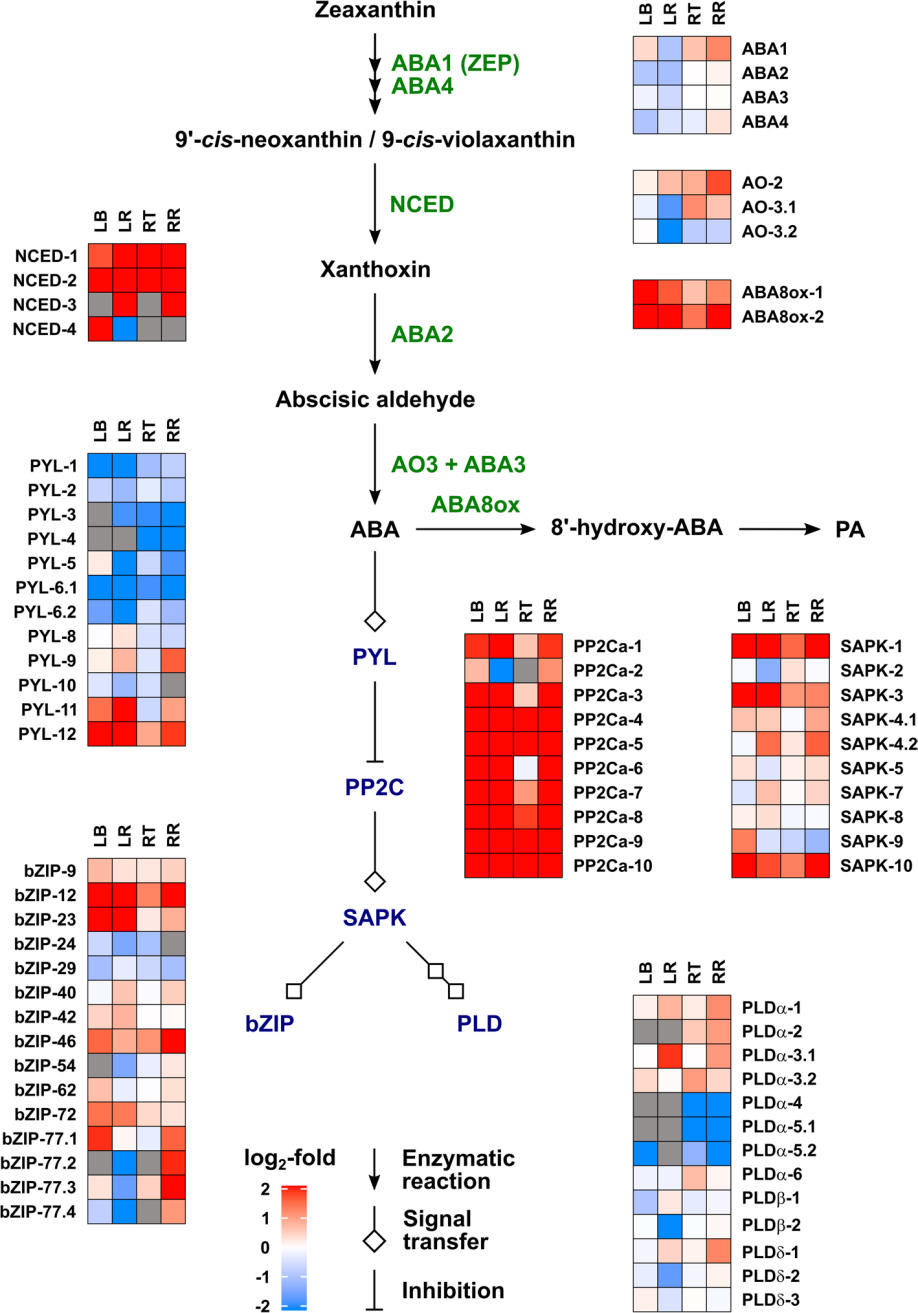
Heat maps for differential expression of genes for ABA metabolism and signalling between watered and droughted wheat seedlings in the 4th leaf base and remaining leaf, and root tip and remaining root. See the legend for Fig. 4 for details

**Fig. 6:**
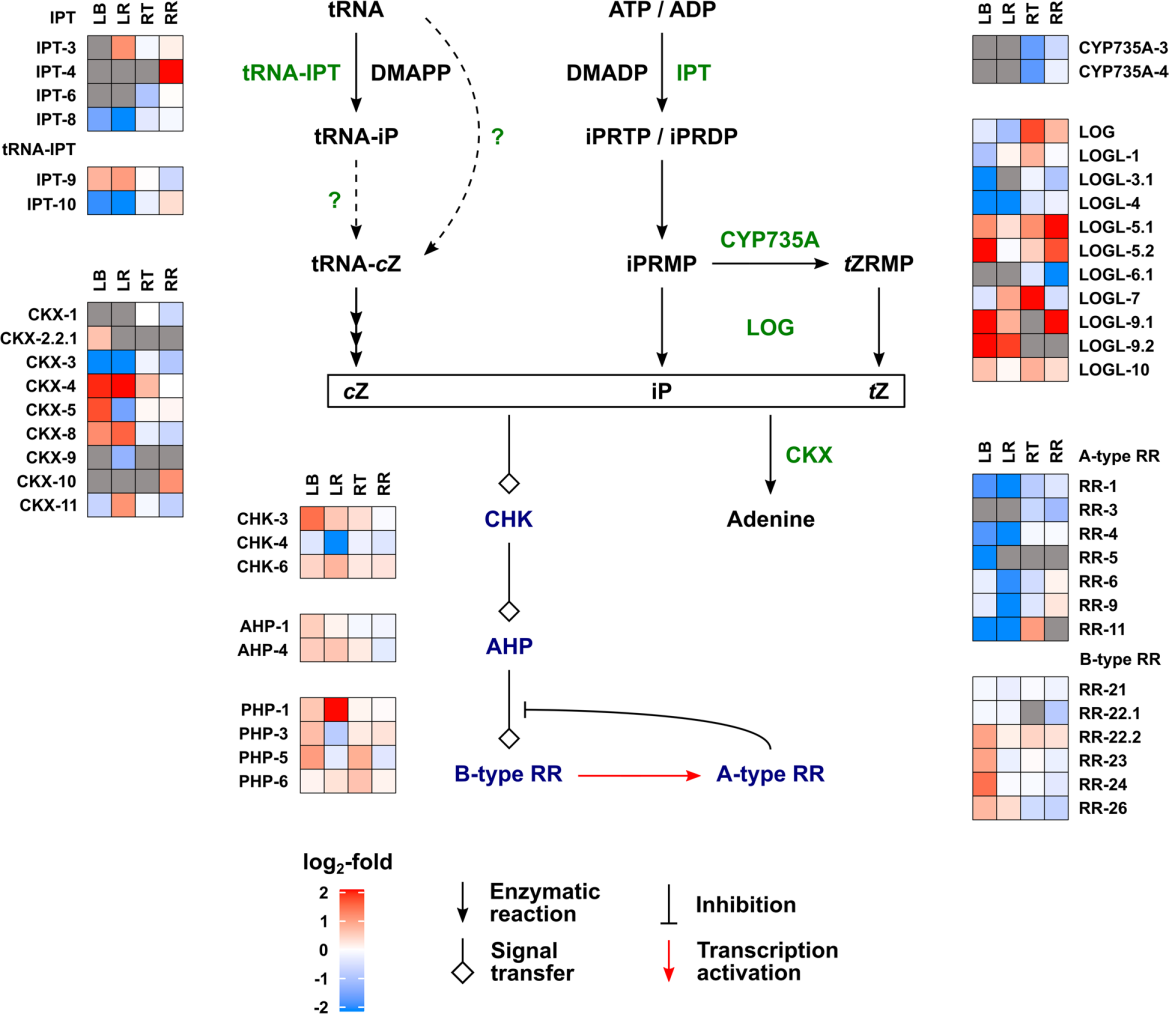
Heat maps for differential expression of genes for cytokinin metabolism and signalling between watered and droughted wheat seedlings in the 4th leaf base and remaining leaf, and root tip and remaining root. See the legend for Fig. 4 for details

**Fig. 7:**
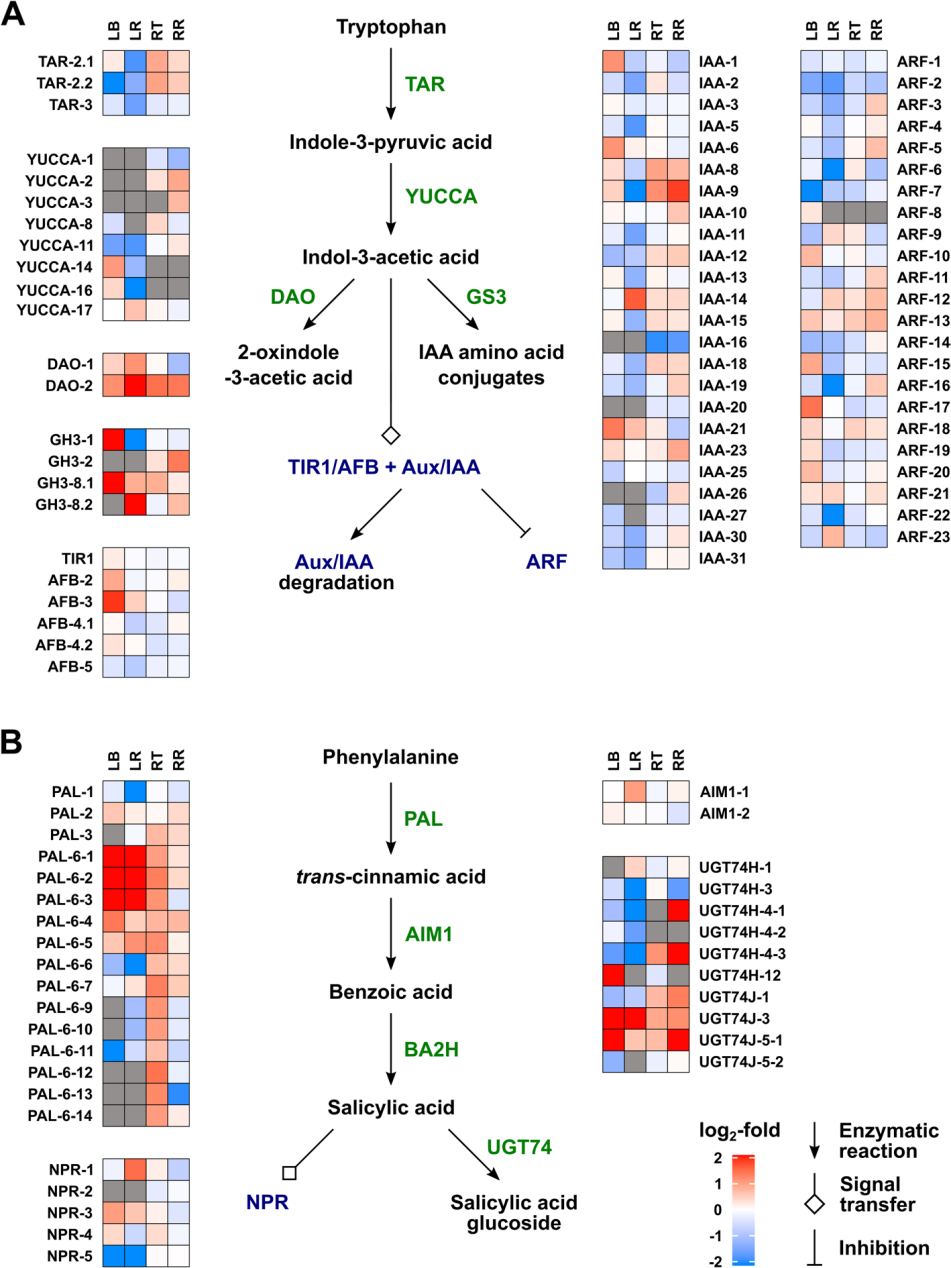
Heat maps for differential expression of genes for auxin (**A**) metabolism and salicylic acid (**B**) signalling between watered and droughted wheat seedlings in the 4th leaf base and remaining leaf, and root tip and remaining root. See the legend for Fig. 4 for details

**Fig. 8:**
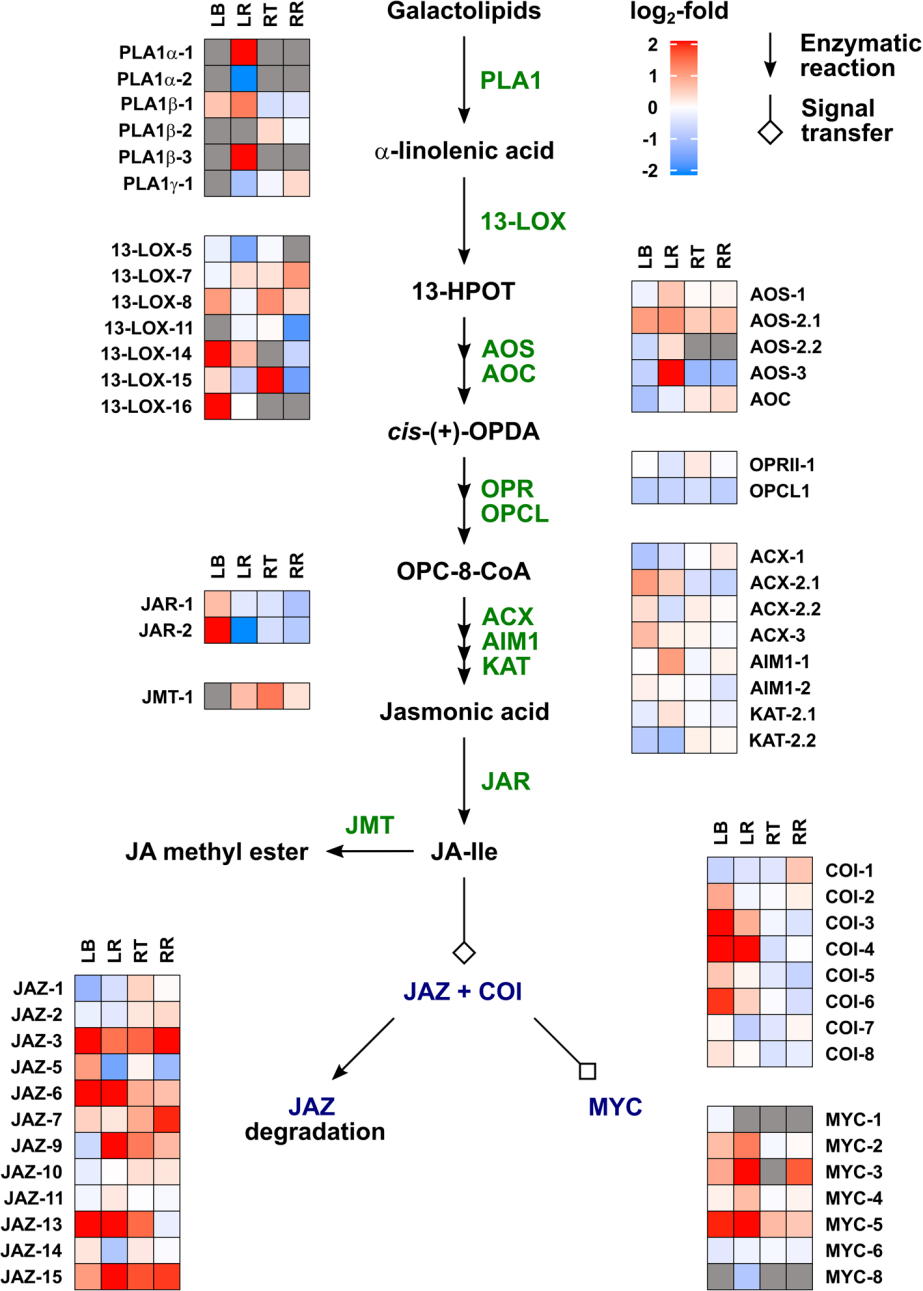
Heat maps for differential expression of genes for jasmonate metabolism and signalling between watered and droughted wheat seedlings in the 4th leaf base and remaining leaf, and root tip and remaining root. See the legend for Fig. 4 for details

Metabolism and signalling genes for the other analysed hormones were curated for wheat and their normalised reads are provided for the four tissue types under well-watered and droughted conditions in Additional file 7, with their differential expression displayed as heat maps in Figs. 5, 6, 7 and 8. Although levels of ethylene, brassinosteroids and strigolactones were not determined, for completeness expression data for their pathway genes are included in Additional file 7.

Differential expression of genes involved in ABA metabolism and signalling is displayed in Fig. 5. Two of four *NCED* genes encoding 9-*cis*-epoxycarotenoid dioxygenases were up-regulated in all tissues by drought, while *NCED-3A* was up-regulated in the remaining leaf and remaining root. Of the other ABA-biosynthetic genes, two *AAO3* genes encoding abscisic aldehyde oxidase were up-regulated by drought only in the roots. *ABAox1* and *ABAox2* encoding CYP707A (ABA 8′-hydroxylases) that catalyze ABA catabolism were up-regulated by drought in the leaf, but not significantly in the root. Genes encoding the ABA signalling components A-type 2C protein phosphatases (PP2CAs) were strongly up-regulated by drought in all tissues as were two *TaSAPK* genes encoding stress/ABA-activated protein kinase that acts downstream of PP2CA. Several of the *PYL* (ABA receptor) genes were down-regulated by drought, while two subfamily I *PYL* genes, *PYL11* and *PYL12* were up-regulated, particularly in the leaf.

For the other hormones, differential gene expression in response to drought was more evident in the leaf tissues than in the roots, even though the levels of these hormones responded similarly to drought in both organs. The data for CKs are present in Fig. 6. Transcripts for the isopentenyltransferase genes *IPT3 – IPT8*, thought to be responsible for *t*Z biosynthesis [54], were present at very low levels in the leaf, with the most abundant, *IPT8*, down-regulated by drought. Although biosynthesis of *c*Z is not fully understood, there is evidence for the involvement of tRNA-IPTs [55]. These are encoded by *IPT9* and *10*, which were expressed at relatively high levels in the leaf tissues, with *IPT10* down-regulated by drought. The isopentenyladenine hydroxylase (CYP735A) genes were not expressed in the leaf, while a number of LOGL genes, encoding CK riboside 5′-monophosphate phosphoribohydrolases [56], were expressed in the leaf, with several down-regulated by drought. Expression of CK dehydrogenase genes (*TaCKX*), responsible for CK catabolism, was modified by drought in the leaf tissues, either negatively or positively, but not in the roots. Type A CK response regulator genes were generally down-regulated by drought in the leaf. With the exception of *TaCOGT-D1.1*, which was strongly up-regulated in the remaining leaf, expression of *O*-glucosyltransferase genes were generally down-regulated by drought in the leaf or unchanged in the root tissues. The IAA-biosynthetic genes *TaTAR2.1* and *TaTAR3.1*, encoding tryptophan aminotransferases and *TaYUCCA11*, encoding a flavin-containing monooxygenase, were slightly down-regulated by drought in the remaining leaf as were AUX/IAA- and ARF-encoding genes involved in auxin-signal transduction (Fig. 7A), consistent with altered IAA signalling in the droughted leaf. Several *PAL* genes, encoding phenylalanine ammonia-lyase, potentially involved in SA biosynthesis, were differentially regulated under drought in the leaf with smaller changes in the root (Fig. 7B). Notably, a UDP-glucosyltransferase gene *UGT74J3* was strongly up-regulated by drought, while the more highly expressed *UGT74H* genes were down-regulated in the remaining leaf. In rice, these glucosyltransferases have been shown to conjugate SA and regulate its concentration [57, 58]. While there was little uniform change in expression of jasmonate biosynthetic genes in response to drought, several genes encoding COI receptors, the JAZ signalling component and MYC transcription factors were up-regulated by drought in the leaf tissues (Fig. 8).

## Discussion

Redistribution of growth between shoots and roots is a common consequence of water restriction [59, 60]; reduced shoot growth moderates water use, while promoted or maintained root elongation maximizes exploitation of the available water. Due to the importance of GAs in regulating growth, this class of hormone is a prime candidate in this growth redistribution, particularly since shoots are much more sensitive to changes in GA concentration than are roots [43]. After five days of progressive water restriction, growth of the elongating third and fourth leaves of the wheat seedlings was significantly reduced, while there was no significant difference in root length (Fig. 1). The growth reduction of the fourth leaf corresponded with substantial decreases in the concentrations of the bioactive GA_1_ and GA_4_ in the base of the elongating leaf (Fig. 2), while there was no change in the remaining leaf tissue, containing the upper leaf sheath and blade. In the root tip, containing the growing region there were small but significant increases in the concentrations of these GAs consistent with the maintained root growth under water restriction. In order to understand the mechanisms for the drought-induced changes in GA content, RNA-seq was used to determine differential gene expression under drought for the four tissue types. Abiotic stress has been shown to induce increased expression of *GA2ox* genes [13] with evidence from work with Arabidopsis and tomato that this is at least partly responsible for the stress-induced physiological response [33, 36, 40, 41, 61]. In both sections of the droughted fourth leaf, there was strong up-regulation of *TaGA2ox4*, with *TaGA2ox3* and *TaGA2ox10* also up-regulated in the remaining leaf, but not in the lower leaf sheath. Indeed, *TaGA2ox3* transcript was present at lower levels in the leaf base in response to drought, potentially in response to the reduced GA content through the homeostasis mechanism, suggesting its expression is not directly regulated by water stress. Potentially through the same mechanism, there was strong drought-induced up-regulation of the GA-receptor gene *GID1* in the leaf tissues, although a response to reduced GA signaling is less clear for the remaining leaf in which the GA content was unchanged. More detailed information on the distribution of GA and gene expression along the leaf may be required to understand the relationship between gene expression and GA content. In the root, *TaGA2ox3* was the most-highly expressed *GA2ox* gene, but was not differentially expressed under drought, as was no other *GA2ox* gene. There are recent reports for maize and rice of up-regulation of *GA2ox* genes in shoots and down-regulation of these genes in roots in response to osmotic stress, indicating tissue-specific differential regulation of these genes [62, 63].

Despite the up-regulation of *GA2ox* genes in the leaf, the concentrations of GA catabolites and precursors are not consistent with higher rates of 2-oxidation as the major cause of the reduction in bioactive GA levels in the leaf base under drought. Indeed, the levels of the 2β-hydroxyGAs, GA_8_, GA_34_, GA_29_ and GA_51_ were also reduced. The concentrations of precursor GAs in both the 13-hydroxy and 13-deoxy pathways to GA_1_ and GA_4_, respectively, were also reduced suggesting a general reduction in GA biosynthesis. Transcripts levels for the biosynthesis genes *GGPPS1*, *KAO* and *GA3ox2* were reduced by water restriction in the remaining leaf section, but less so in the base of the leaf sheath, in which nevertheless GA levels were reduced. Overall, under well-watered conditions transcripts for most biosynthetic genes were more abundant in the remaining leaf than in the leaf base, despite the latter containing higher concentrations of bioactive GA_1_ and GA_4_ as is predictable for the elongation zone. The exception is the *GA3ox2* transcript, which was equally distributed between the two leaf sections. These results are in agreement with those in [64], in which the distribution of GAs and expression of GA-biosynthesis genes were determined along a growing maize leaf. They showed that bioactive GAs and *GA3ox* expression peaked at the boundary between the division and cell elongation zones, while precursors and transcripts encoding enzymes for earlier biosynthetic steps were abundant in the more mature part of the leaf. Most strikingly, transcript reads for *KAO* were very low in the leaf base, as was the case also for the maize leaf [64]. The data would suggest that the early stages of biosynthesis occur predominantly in the remaining leaf, containing the leaf blade, with GAs and/or precursors transported to the base. The leaf blade may experience more stress under drought, although this did not translate into reduced GA content in the remaining leaf. However, this material is heterogeneous and it will be necessary to determine the effect of drought on GA distribution.

Growth and its response to water limitation are regulated by the combined activity of multiple hormones [47, 48], so we took the opportunity to measure the concentrations of ABA, CKs, IAA, JA, SA and associated metabolites (Fig. 3 and Additional file 2) and to determine the effect of drought on expression of genes involved in their metabolism and signaling pathways (Figs. 5, 6, 7 and 8 and Additional file 7). In contrast to GAs, ABA levels were strongly increased by water restrictions in both leaf and root samples, accompanied by up-regulation of genes encoding NCED and ABA8ox metabolism enzymes and the PP2C signaling components, as has been previously reported [65–70]. Several of the *PYL* (ABA receptor) genes were down-regulated by drought, as has been shown for the subfamily II *PYLs* (*PYL4*, *PYL5* and *PYL6*) in rice [71]. These genes are also down-regulated by ABA, consistent with their involvement in ABA homeostasis [72]. Two subfamily I *PYL* genes, *PYL11* and *PYL12* were up-regulated, particularly in the leaf, as reported by [71].

The levels of the other analysed hormones responded similarly to drought in the leaf and roots, although expression of the genes monitored for their metabolism and signal transduction changed much more in the leaf tissues than in the roots. The very substantial reduction in the levels of *t*Z and its riboside in the leaf sheath base and root tip in response to water restriction is consistent with reduced leaf growth and maintained root elongation, which is inhibited by CK signalling [73, 74]. The reduced *t*Z and its riboside was accompanied by increased levels of their *O*-glucosides suggesting enhanced conjugation, although, with the exception of *TaCOGT-D1.1*, there was limited indication of increased expression of *O*-glucosyltransferase genes. Of the *IPT* genes thought to be responsible for *t*Z biosynthesis [54], only *IPT8* was expressed in the leaf and was down-regulated by drought. The reduced expression of *CKX* under drought is in agreement with previous reports [75], but was observed only in the leaf. The lack of a clear correlation between changes in CK levels and expression of metabolism genes in rice in response to drought was noted in [76], in which it was suggested that drought affects CK distribution and degradation. In contrast to those of *t*Z and its riboside, *c*Z and *c*ZR levels increased in the leaf base and root tip under drought. *c*Z is thought have a role in regulating growth under stress [77, 78], and its increase is consistent with a stress response, although this is not correlated with growth restriction in the root. Unexpectedly, there was no change in IAA content in the leaf or root in response to drought, despite previous reports of reduced IAA under such conditions, for example [76]. However, higher levels of 2-oxindole-3-acetic acid in all tissues and of IAA-glutamate in the leaf base indicate increased IAA turnover. Furthermore, RNA-seq revealed down-regulation of genes encoding Aux/IAA and ARF proteins, consistent with altered IAA signal transduction in the droughted leaf. A role for jasmonate in drought responses is well documented [79–81], with reports of water restriction resulting in increased jasmonate levels [82]. However, in the current experiment, the levels of JA and JA-isoleucine were lower in the droughted organs, although expression of several *JAZ*, *COI* and *MYC* genes encoding components of JA signal transduction was up-regulated in the droughted leaf. SA levels were also reduced by drought in the leaf base and root tip, although they were higher in the remaining leaf, suggesting a redistribution under drought. Munoz-Espinoza et al. [80] reported transient increases in the levels of SA and JA in roots and shoots of tomato plants under water restriction, while ABA levels increased progressively. Their results indicated that ABA reduced the production of SA and JA as the drought progressed, resulting in lower levels of these hormones in prolonged drought, as found in the current study.

## Summary and conclusions

Growth restriction in an elongating leaf as a result of progressive drought was associated with a reduction in bioactive GA concentration in the leaf base, which contains the growing zone of the sheath, while small increases in bioactive GAs in the root tip under drought were consistent with maintained root elongation. These changes were not evident in the remaining leaf and root and were thus associated specifically with the growth of these organs. While drought resulted in up-regulation of *GA2ox* gene expression in the leaf, in particular of *TaGA2ox4*, quantitative analysis of GA catabolites and biosynthetic intermediates indicated that reduced biosynthesis rather than increased inactivation explained the reduction in GA content. Genes encoding several GA-biosynthetic enzymes were down-regulated by drought in the leaf, but not in the root. These changes occurred throughout the leaf suggesting that the distribution of GAs and precursors may be an important factor in the response to the stress. ABA and transcripts for *NCED* biosynthesis genes and genes encoding type-A PP2C signalling components were substantially increased in all tissues by drought indicating that the stress was perceived throughout the plant, although overall differentially expressed genes were more abundant in the leaf tissues compared with roots. The concentrations of *t*Z and its riboside were higher in the leaf base and root tip than in the remaining organs and were very highly attenuated under drought, with increased concentrations of their *O*-glucosides. In contrast, *c*Z and *c*ZR concentrations increased in the leaf base and root tip under drought, in support of their suggested role in stress responses, whereas the *trans* isomers are associated with growth. The concentration of IAA was unchanged, while those of JAs and SA were reduced under drought for most tissues, potentially due to the prolonged and progressive nature of the stress. For most of the hormones investigated it was not possible to correlate the changes in hormone abundance with expression of metabolism genes, and more detailed knowledge of their distribution and its drought-induced changes will be required.

## Methods

### Plant growth conditions and sampling

Seeds of *Triticum aestivum* cv. Cadenza were imbibed on wet filter paper at 4 °C for 2 d in the dark and then germinated at room temperature for a further 2 d. Two uniformly germinated seedlings were planted in pots measuring 13x13x12 cm containing 500 g of dried field soil (loamy sand: 88% sand, 5% silt, 7% clay) and 1.5 g of Osmocote® fertilizer (Sierra Chemical), which had been allowed to take up water to full capacity (0.26 g tap water per 1 g of soil). One of the seedlings was removed after 4-5 d. The soil water content was maintained at full capacity for 7-8 days until the 3rd leaf was visible, after which watering of the droughted group was discontinued, while the control group was watered daily as before. The plants were grown in a controlled environment cabinet at 22/18 °C and 70/80% relative humidity (day/night) with a 14-h photoperiod and light flux of 150 μmol·m^− 2^·s^− 1^. The length of each visible leaf was recorded daily. After 5 days of drought, the 4th leaf from both sets of plants was harvested after removing the outer leaves and dissected into the bottom 3-cm of the sheath and the remaining leaf (Additional file 1; Fig. S1), which were frozen in liquid N_2_. The roots were washed briefly in water to remove soil, dried on a paper towel and the length of the longest root measured. 3-cm of the tips of the major roots (seminal and nodal) were dissected (Additional file 1; Fig. S1) and frozen in liquid N_2_, as were the remaining roots.

### Evaluation of stress

The relative water content was measured on the 3rd leaf using the protocol in [83]. Free proline content was determined according to [84] with slight modifications. Homogenized leaves (100 mg) were incubated in 3 ml of 3% sulphosalicylic acid at 96 °C for 10 minutes. Samples were clarified by centrifugation and 1 ml of supernatant was mixed with 2 ml of 50% acetic acid, 2 ml of 2.5% acidic ninhydrin solution and boiled for another 30 min. The reaction product was liquid-liquid extracted by 5 ml of toluene and the absorbance of the toluene fraction was measured at 520 nm. The concentration of proline was determined using a standard curve (0-30 μg) and expressed as μg·mg^− 1^ of protein. Estimation of protein concentration was according to [85] by spectrophotometric measurement of absorbance of PBS (100 mM, pH 7.8) buffered leaf extracts at 260 and 280 nm. The level of lipid peroxidation was determined by measuring the MDA concentration as described in [86]. Gas exchange measurements were made on the 2nd leaf using the LI-COR 6400-XT infrared gas analyzer with attached leaf chamber LI6400-40 (Li-COR, Biosciences). The measurements were performed with a CO_2_ reference concentration of 400 μmol·mol^− 1^, an air flow of 200 μmol·s^− 1^, block temperature of 20 °C, photosynthetic photon flux density of 1800 μmols·m^− 2^·s^− 1^ and relative humidity between 55 and 65%.

### Hormone quantitative analysis

For analysis of GAs, dissected leaf and root sections from watered and droughted plants (harvested as described above) were combined from 3 plants for each of 3 biological replicates for each tissue type and treatment and then frozen in liquid N_2_. The samples were freeze-dried, ground to a powder, from which 10 mg was extracted in the presence of 3 pmol ^2^H_2_-labelled GA internal standards (OlchemIm s.r.o., Olomouc, Czech Republic), purified and analysed by UHPLC-MS-MS as described in [87] using a Xevo TQ-XS triple quadrupole mass spectrometer (Waters Milford, MA, USA). For the global hormone analysis, samples were prepared in the same way with 7 biological replicates and 3 mg sample extracted, purified and analysed as described in [88].

### Analysis of gene expression by qRT-PCR

Total RNA was extracted from three biological replicates of plant tissues using E.Z.N.A.® Plant RNA kit (Omega Bio-Tek) and treated with RNase-free DNase I (Promega). cDNAs were synthesized by RevertAid H Minus reverse transcriptase using oligo (dT)18 primers (Thermo Fisher Scientific). The subsequent qRT-PCR analysis of each cDNA was performed in three technical replicates on the CFX96 Real-Time System with a C1000 thermal cycler (Bio-Rad). The reaction mixture contained 400 nM of each primer (Additional file 1: Table S2) and Xceed qPCR SYBR Green I mix (Institute of Applied Biotechnologies, CZ; discontinued). PCR efficiencies of primer pairs were determined using a dilution series of cDNA and standard curve method. The wheat α-tubulin gene (*TaTUBa*, GenBank accession number U76558) and a gene with GenBank accession number CJ705892 [89] were used as reference genes. Normalization of relative gene expression was performed with respect to primer amplification efficiency and the internal control genes as described by [90], without taking into account the error propagation from amplification efficiency determination.

### RNA sequencing and differential gene expression analysis

Total RNA samples from the four sample groups were isolated using the Monarch Total RNA Miniprep Kit (New England Biolabs Inc., Ipswich, MA, USA) according to the manufacturer’s protocol. The RNA quantity and integrity were measured with an Agilent 2100 Bioanalyzer (Agilent Technologies, Santa Clara, CA, USA). Samples with RNA integrity number (RIN) higher than 6.3 were used in the RNA sequencing (RNA-seq) which was performed at Novogene Co. Ltd. (Beijing, China) on the Illumina NovaSeq 6000 platform with a paired-end 150 bp sequencing strategy. The raw data were further processed, and low-quality bases were removed using Trimmomatic-0.39 [91]. This included Illumina adapter sequences, bases with Qscore < 13 and reads shorter than 36 bases. Cleaned reads were mapped to the reference genome of *T. aestivum* cv. Chinese Spring, IWGSC RefSeq1.1^1^ using HISAT2-2.1.0 [92] with default parameters. Reads count matrix was generated using only uniquely mapped reads with Rsubread-2.0.1 (featureCounts function) [93]. Differential gene expression analysis was performed using DESeq2 software [94]. LFC estimates were shrunken using the ashr method within the lfcShrink function. Only genes with FDR-adjusted *p* values < 0.05 (Benjamin-Hochberg procedure) and LFC of ≥1 or ≤ − 1 (2-fold change) were considered as differentially expressed. Hierarchical cluster analysis (HCA) and principal component analysis (PCA) was performed after regularized-logarithm transformation (rlog) of count data. For the heat maps in Figs. 4, 5, 6, 7 and 8 the normalized reads for the homoeologues were combined and mean values for three biological replicates were calculated. The LFC was calculated using the formula: LFC = log_2_(B) - log_2_(A), where A and B are the means of summed reads for watered and droughted samples, respectively. The number of normalized reads and LFCs for individual homeologues together with the gene IDs are provided in Additional file 7. The heat maps were generated using pheatmap-1.0.12 [95] R package and the pathways were drawn using Inkscape 1.1. The heat map in Additional file 1 : Fig. S3A was generated using ComplexHeatmap-2.2.0 R package [96]. Volcano plots were generated using the EnhancedVolcano R package [97].

### Gene ontology (GO) enrichment analysis

The functional enrichment analysis was performed using g:Profiler and gprofiler2 R package (version e104_eg51_p15_3922dba) [98] with FDR < 0.05 and LFC of ≥1 or ≤ − 1 for up-regulated and down-regulated genes, respectively.

## Supplementary Information

**Additional file 1: Table S1.** Effect of water restriction on relative water content, MDA and proline concentration, photosynthetic rate and gas exchange. **Table S2.** Sequences of primers used for qRT-PCR analysis. **Figure S1.** Schematic diagram of a wheat seedling, indicating the tissue sections harvested for analysis. **Figure S2.** Volcano plots of differentially expressed genes in each tissue type. **Figure S3.** Hierarchical clustering heatmap, principal component analysis and tissue distribution of differentially expressed genes. **Figure S4-Figure S7.** Gene ontology analysis for biological function of differentially-regulated genes in the leaf base (Fig. S4), remaining leaf (Fig. S5), root tip (Fig. S6) and remaining root (Fig. S7). **Figure S8.** Comparison of qRT-PCR and RNA-seq for determination of differential expression of selected genes between watered and droughted plants.

**Additional file 2.** Concentrations of hormones (ABA, CKs, IAA, JAs, SA acid and related metabolites in each tissue type in watered and droughted wheat seedlings.

**Additional file 3.** Lists of mapped genes from RNA-seq for the 4th leaf base (File 3).

**Additional file 4.** Lists of mapped genes from RNA-seq for the 4th remaining leaf (File 4).

**Additional file 5.** Lists of mapped genes from RNA-seq for the 4th root tip (File 5).

**Additional file 6.** Lists of mapped genes from RNA-seq for and remaining root (File 6) in order of differential expression.

**Additional file 7.** Normalised reads for wheat hormone metabolism and signalling genes from RNA-seq for each tissue type under watered or droughted conditions.

## Data Availability

The datasets supporting the conclusions in this article are included in the article and its additional files.

## Abbreviations

ABA: abscisic acid
CK: cytokinin
CKX: cytokinin dehydrogenase
CPS: *ent*-copalyl diphosphate synthase
DEG: differentially expressed gene
GA: gibberellin
GA20ox: GA 20-oxidase
GA2ox: GA 2-oxidase
GA3ox: GA 3-oxidase
GGPPS: *trans*-geranylgeranyl diphosphate synthase
GID: GIBBERELLIN INSENSITIVE DWARF
GO: gene ontology
IAA: indole-3-acetic acid
JA: jasmonate
KAO: *ent*-kaurenoic acid oxidase
KO: *ent*-kaurene oxidase
KS: *ent*-kaurene synthase
LFC: log_2_-fold change
qRT-PCR: quantitative reverse transcription-polymerase chain reaction
SA: salicylic acid
*cZ*: *cis*-zeatin
*c*ZR: *cis-*zeatin riboside
*t*Z: *trans*-zeatin
*t*ZR: *trans*-zeatin riboside
UHPLC-MS-MS: ultra-high performance liquid chromatography tandem mass spectrometry
