# Supplementary material for: Changes in the concentrations and transcripts for gibberellins and other hormones in a growing leaf and roots of wheat seedlings in response to water restriction
Source: BMC Plant Biol. 2022 Jun 9;22:284. doi: 10.1186/s12870-022-03667-w (PMC9178827; doi:10.1186/s12870-022-03667-w)
Supplement: Supplementary file 1 — Additional file 1: Table S1. Effect of water restriction on relative water content, MDA and proline concentration, photosynthetic rate and gas exchange. Table S2. Sequences of primers used for qRT-PCR analysis. Figure S1. Schematic diagram of a wheat seedling, indicating the tissue sections harvested for analysis. Figure S2. Volcano plots of differentially expressed genes in each tissue type. Figure S3. Hierarchical clustering heatmap, principal component analysis and tissue distribution of differentially expressed genes. Figure S4-Figure S7. Gene ontology analysis for biological function of differentially-regulated genes in the leaf base (Fig. S4), remaining leaf (Fig. S5), root tip (Fig. S6) and remaining root (Fig. S7). Figure S8. Comparison of qRT-PCR and RNA-seq for determination of differential expression of selected genes between watered and droughted plants. [file 12870_2022_3667_MOESM1_ESM.pdf]

Table S1. Effect of water restriction on relative water content, MDA and proline concentration (A) and on photosynthetic rate and gas exchange (B).

A

	**RWC**		**MDA content**		**Proline content**	
	%	SD	$nmol.mg\ FW^{-1}$	SD	$\mu g.mg\ protein^{-1}$	SD
Watered	96.1	2.2	578.2	296.0	21.5	2.5
Drought	81.5**	7.5	1198.0*	207.6	101.7*	28.3

B

		**Photosynthetic rate**		**Water conductance**		**Intercellular CO_2 conc.**	
		$\mu mol\ CO_2.m^{-2}.s^{-1}$	SD	$mol.m^{-2}.s^{-1}$	SD	$\mu mol.mol^{-1}$	SD
Day 5	Watered	33.9	4.0	0.39	0.07	236.9	10.7
	Drought	29.9	2.4	0.30	0.04	219.3	8.6
Day 6	Watered	40.3	3.6	0.52	0.06	249.0	9.0
	Drought	30.9*	5.0	0.30*	0.08	208.2*	26.7

*Significantly different from value for watered plants, $p<0.05$; ** $p<0.01$.
RWC, relative water content; MDA, malondialdehyde.

Table S2. Sequences of primers used for qRT-PCR analysis

Primer name	Sequence (5' to 3')
TaTUBα_Fw[1]	CCCTGAGGTTTGATGGTGCT
TaTUBα_R[1]	TGGTGATCTCAGCAACGGAC
EST_CJ705892_Fw[1]	GCCTCAGTGGTAGGAGCATT
EST_CJ705892_R[1]	TTCAGCAAATGCGGTGGTTG
TaGA2ox3_Fw	TGCTAAAACCCGCCTTTCC
TaGA2ox3_R	CAGAAGACCACTTTGATGCC
TaGA2ox4_Fw	GTACAGTAGTGGTAGCACTAGG
TaGA2ox4_R	GCTCCGACACACATCAAAC
TaGA2ox7_Fw	AGTACAAGAGCAGCACCCACAA
TaGA2ox7_R	CGTAACGCACGGAAGCTAGTCT
TaGA2ox10_Fw	TTGGAGGCATCAGGTGAAG
TaGA2ox10_R	CATCTCCATACACACCACATAC
TaGID1_Fw	GATCAAGATACACGGCAACATCCTG
TaGID1_R	CTGTCCTGGAGCGTGACGAA
TaPP2C-1_Fw	GCAAGGTCATACAGTGGAATGGCTACC
TaPP2C-1_R	GAGGAACAATTGTGACCTCTGGGACA

[1] Dudziak et al. Identification of stable reference genes for qPCR studies in common wheat (*Triticum aestivum* L.) seedlings under short-term drought stress. Plant Methods. 2020;16:58.

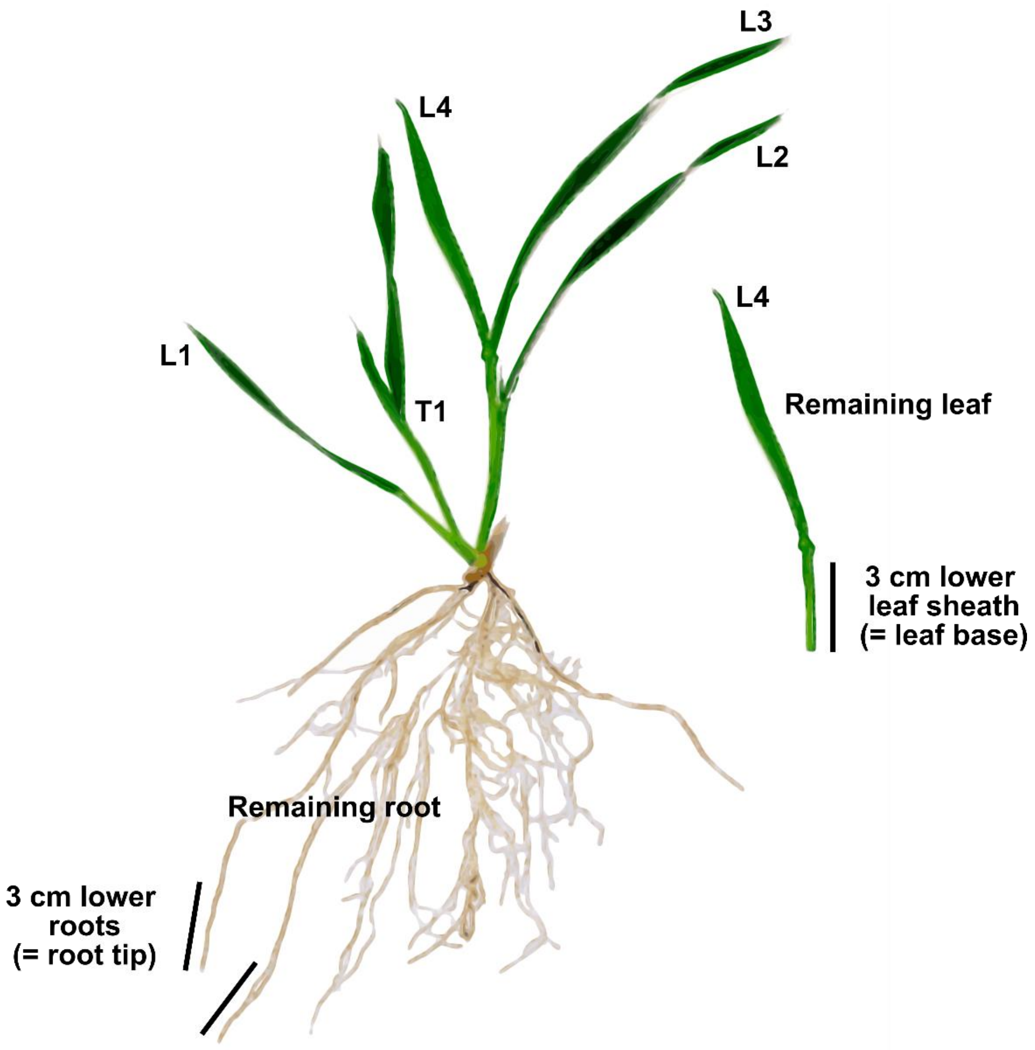

Figure S1. Diagram illustrating the tissue sampling for analysis. After removing the three outer leaves the 4th leaf was removed and divided into the bottom 3 cm of the leaf sheath measured from the node and the remaining leaf. Roots were divided into 3 cm taken from the tip of all major seminal and nodal roots and the remaining roots. L1 - L4 leaves in order of appearance; T1 1st tiller.

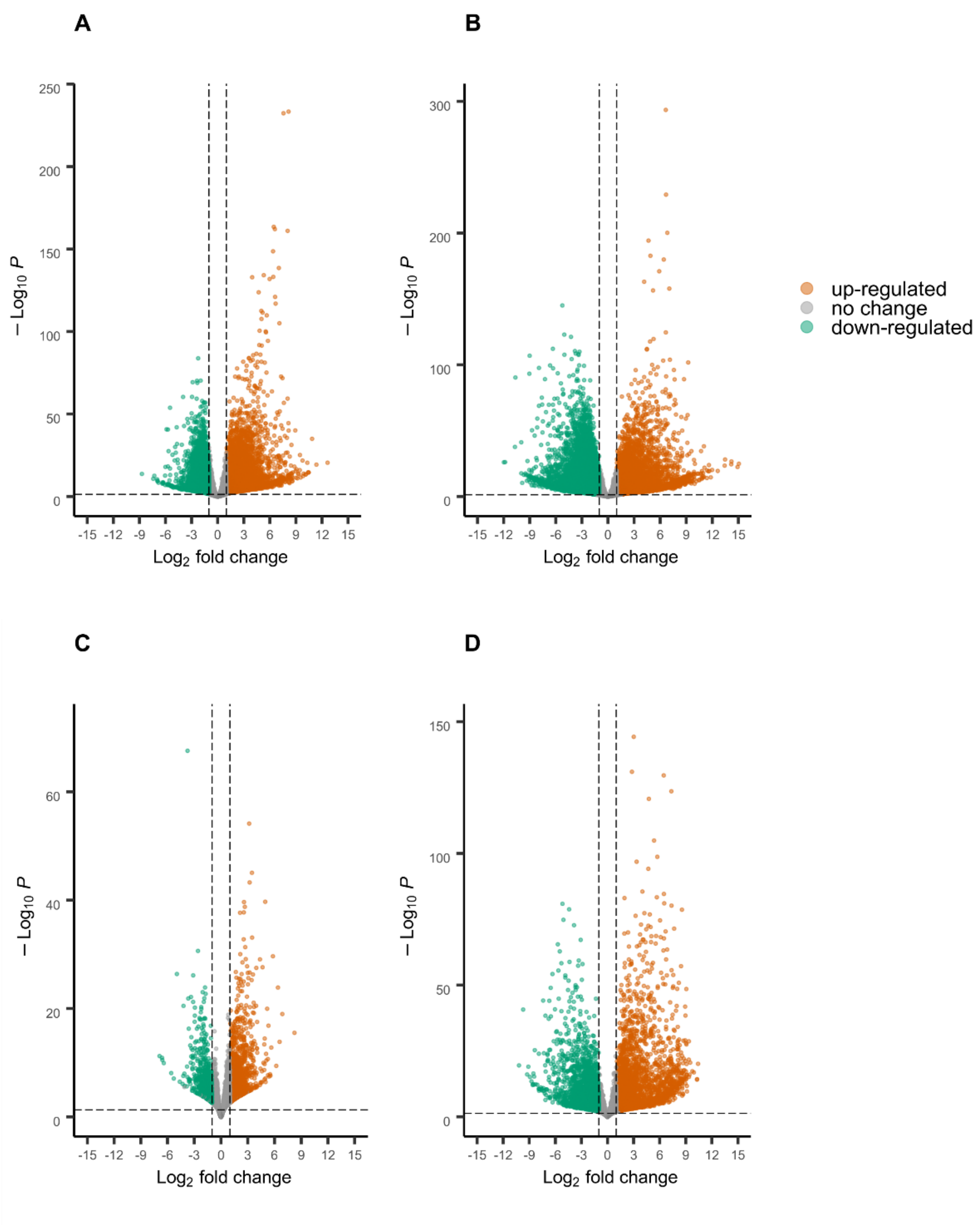

Figure S2. Volcano plots of differentially expressed genes in leaf base (A), remaining leaf (B), root tip (C) and remaining root (D). Orange dots represent up-regulated genes (LFC ≥ 1 and FDR < 0.05), green dots represent down-regulated genes (LFC ≤ -1 and FDR < 0.05), grey dots represent genes that are not significantly differentially expressed. The horizontal dashed line represents FDR cut-off of 0.05 and two vertical lines represent the cut-offs of LFC = -1 and LFC = 1.

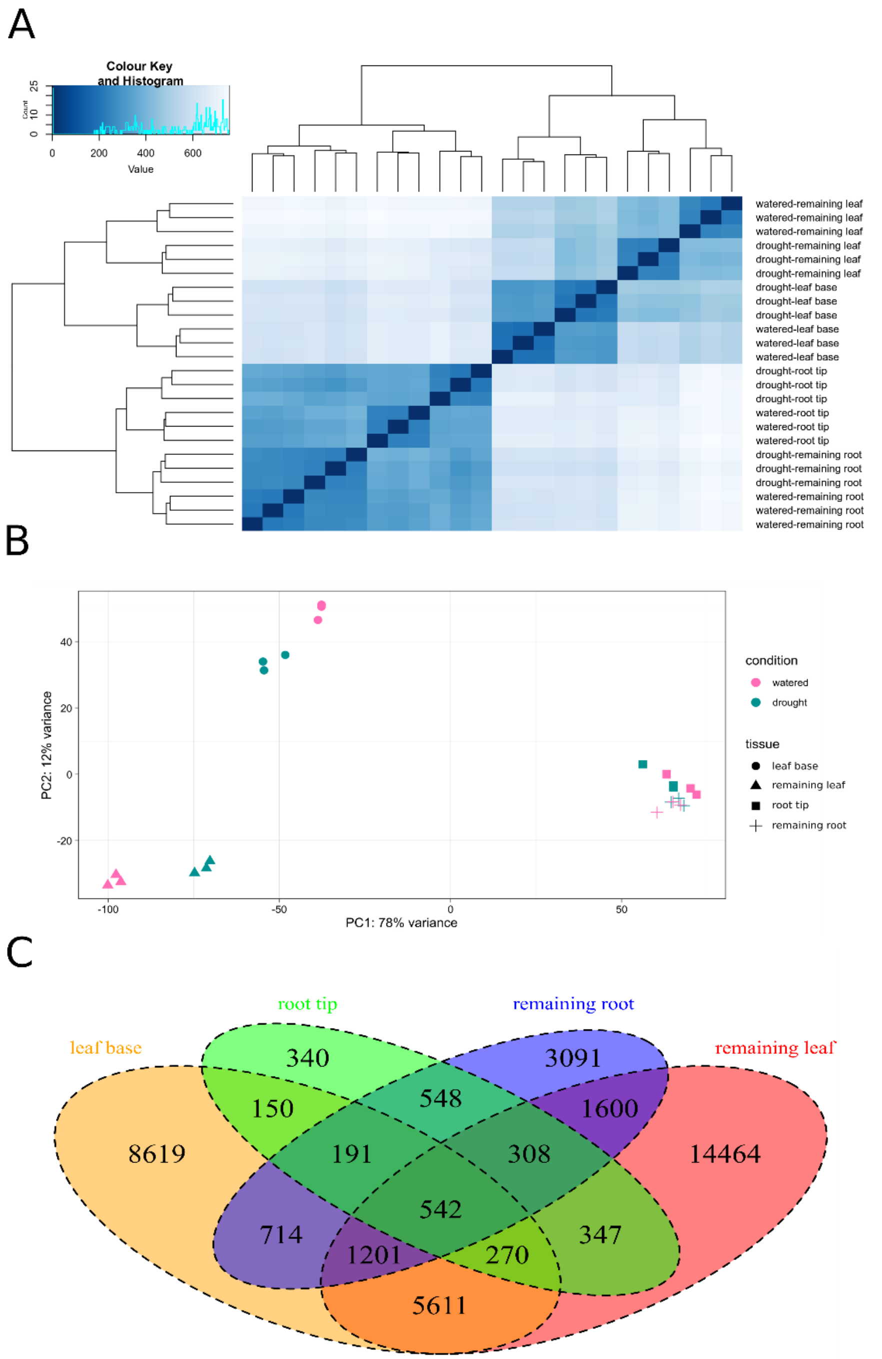

Figure S3. Hierarchical clustering heatmap of sample-to-sample distances using regularised-logarithm transformed values (A). Principal component analysis (PCA) of the transcriptome of watered and droughted samples (B). Venn diagram of differentially expressed genes (DEGs), showing the overlap between DEGs in watered and droughted conditions for all tissue types (C).

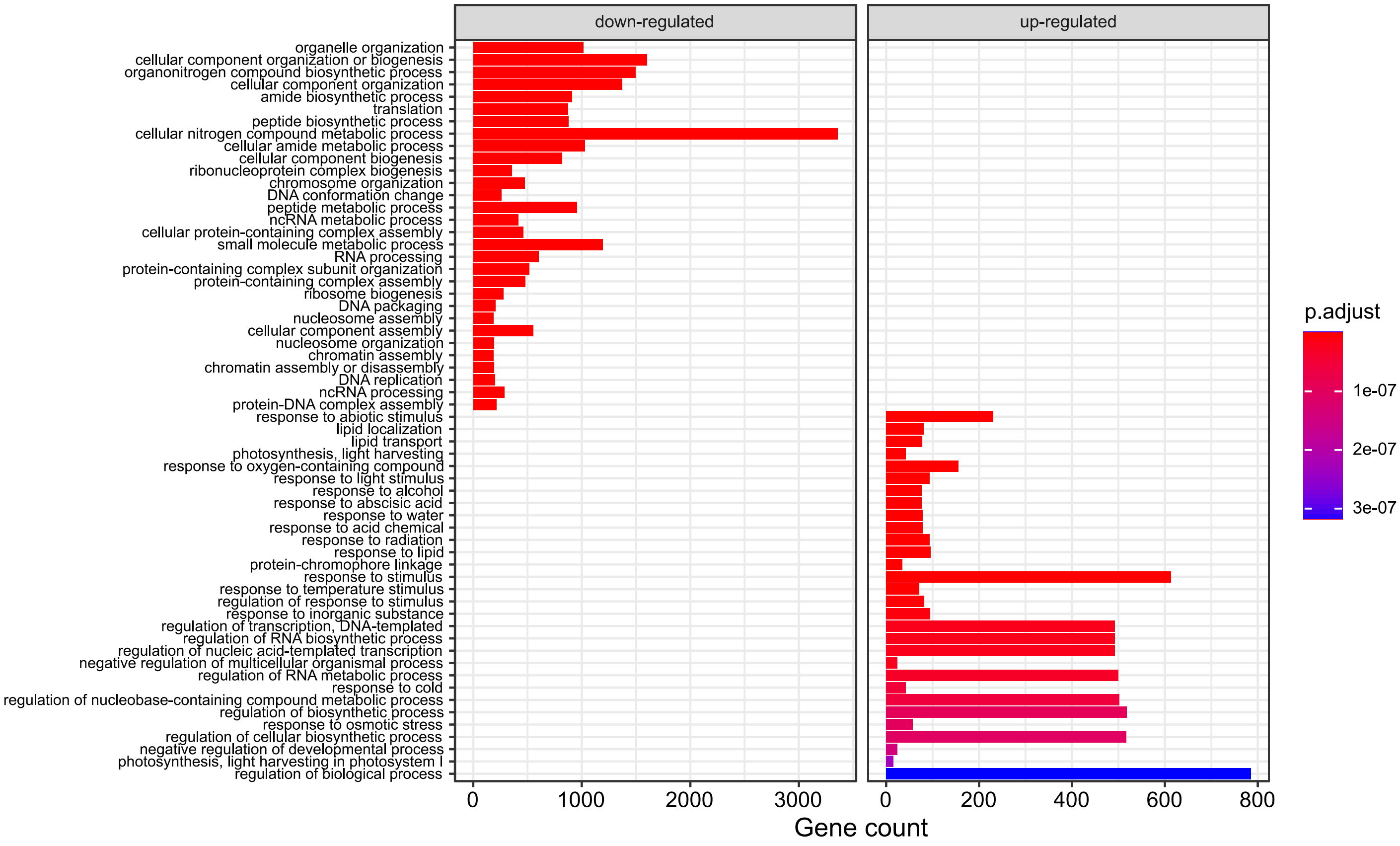

Figure S4. Thirty most significantly enriched biological processes GO terms for up-regulated and down-regulated genes in the leaf base in response to water deficit. The length of the bar indicates the number of genes matching the term. Colour code indicates *p*-values after correction for multiple testing.

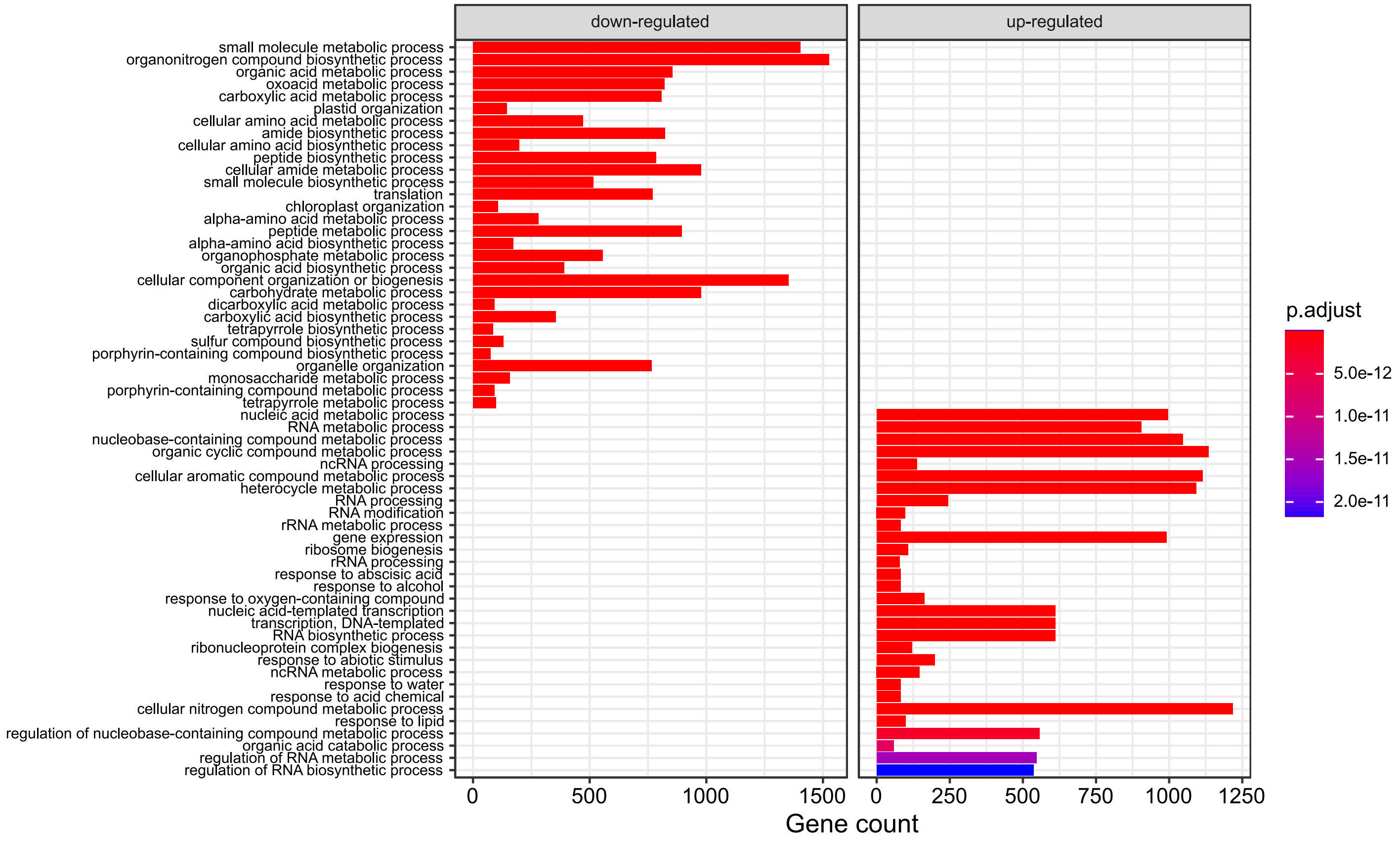

Figure S5. Thirty most significantly enriched biological processes GO terms for up-regulated and down-regulated genes in the remaining leaf in response to water deficit. The length of the bar indicates the number of genes matching the term. Colour code indicates *p*-values after correction for multiple testing.

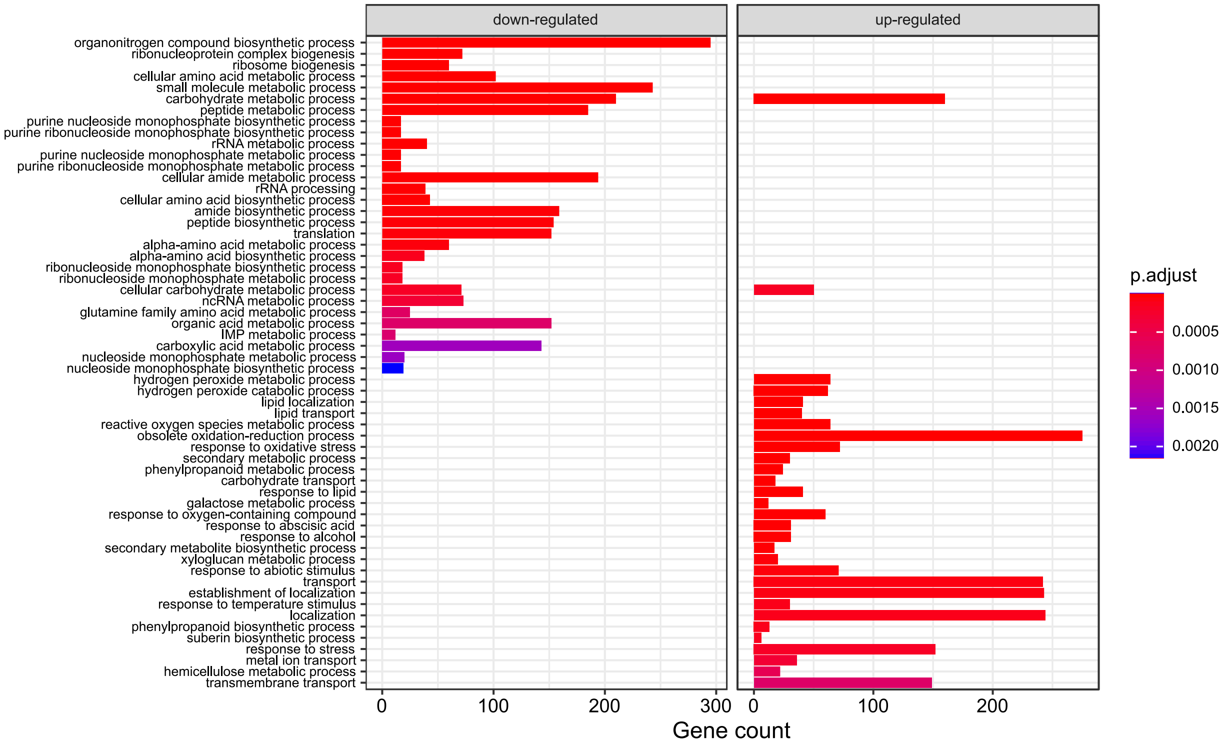

Figure S6. Thirty most significantly enriched biological processes GO terms for up-regulated and down-regulated genes in the root tip in response to water deficit. The length of the bar indicates the number of genes matching the term. Colour code indicates *p*-values after correction for multiple testing.

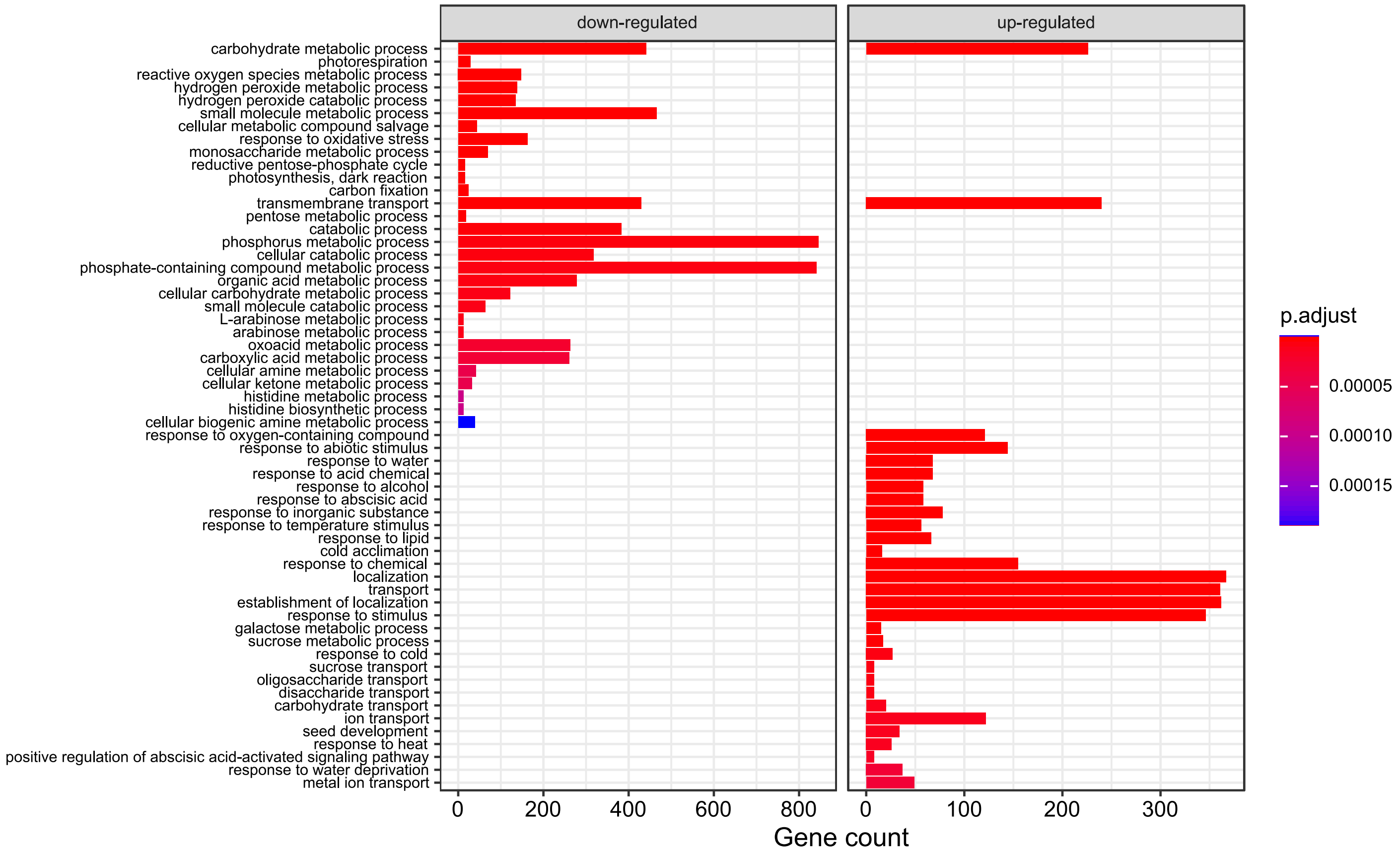

Figure S7. Thirty most significantly enriched biological processes GO terms for up-regulated and down-regulated genes in the remaining root in response to water deficit. The length of the bar indicates the number of genes matching the term. Colour code indicates *p*-values after correction for multiple testing.

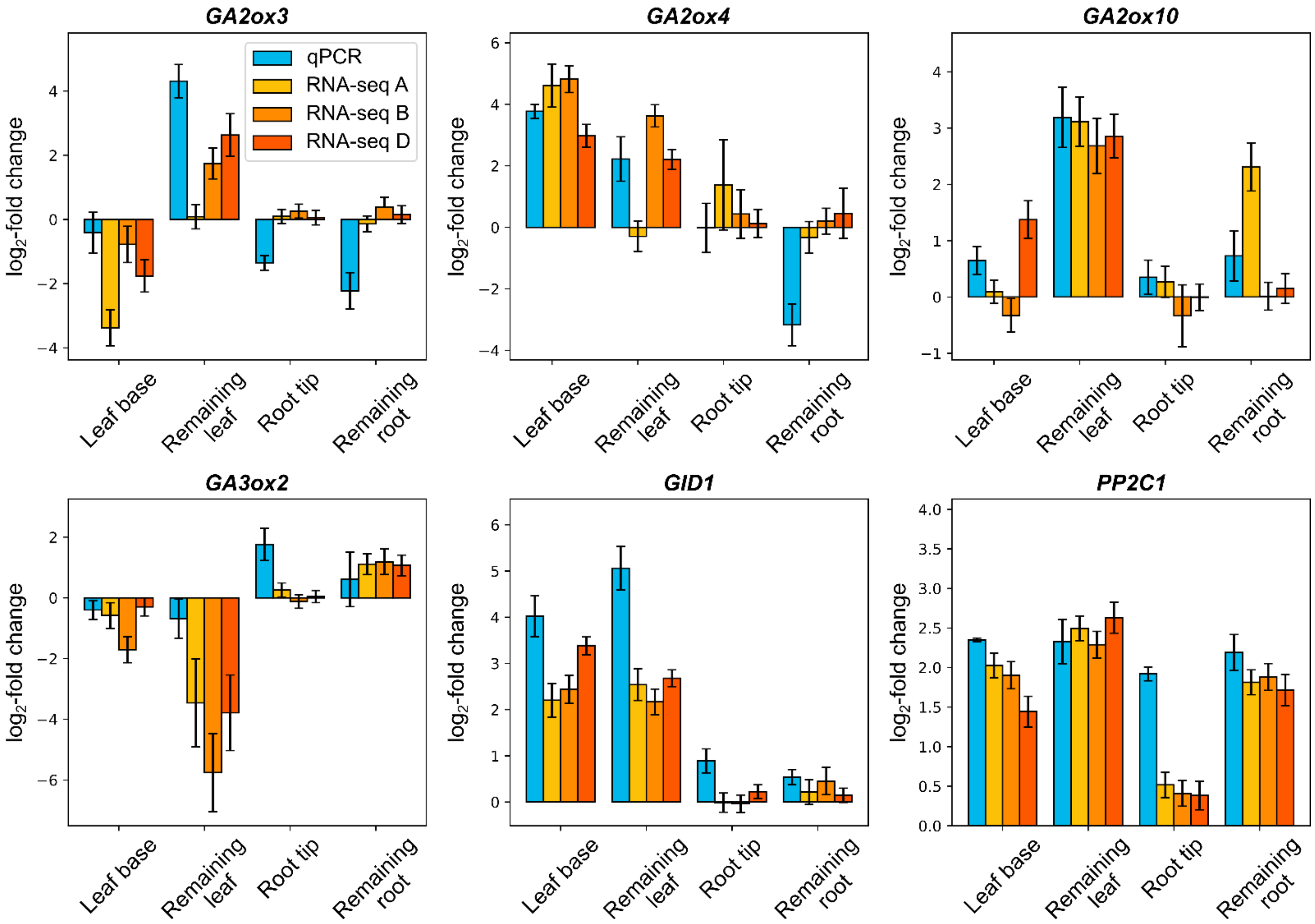

Figure S8. Comparison of qRT-PCR and RNA-seq for determination of differential expression of selected genes between watered and droughted plants. The data show the $\log_2$-fold change drought:watered from separate experiments as the means of three replicates +/- SD. The primers for qRT-PCR were designed to amplify all three homoeologues.
